# Unnatural Amino Acid‐Based Ionic Liquid Enables Oral Treatment of Nonsense Mutation Disease in Mice

**DOI:** 10.1002/advs.202306792

**Published:** 2024-01-30

**Authors:** Yujie Shi, Ningning Shi, Yuelin Yang, Zhetao Zheng, Qing Xia

**Affiliations:** ^1^ State Key Laboratory of Natural and Biomimetic Drugs Peking University Beijing 100191 China; ^2^ Department of Pharmaceutical Analysis School of Pharmaceutical Sciences Peking University Beijing 100191 China; ^3^ Beijing Key Laboratory of Molecular Pharmaceutics and New Drug Delivery Systems Peking University Beijing 100191 China; ^4^ Department of Molecular and Cellular Pharmacology School of Pharmaceutical Sciences Peking University Beijing 100191 China

**Keywords:** ionic liquid, DMD, dystrophin restoration, oral treatment, unnatural amino acid

## Abstract

This investigation addresses the challenge of suboptimal unnatural amino acid (UAA) utilization in the site‐specific suppression of nonsense mutations through genetic code expansion, which is crucial for protein restoration and precise property tailoring. A facile and economical oral liquid formulation is developed by converting UAAs into ionic liquids, significantly enhancing their bioavailability and tissue accumulation. Empirical data reveal a 10‐fold increase in bioavailability and up to a 13‐fold rise in focal tissue accumulation, alongside marked improvements in UAA incorporation efficiency. A 4‐week oral administration in *mdx* mice, a model for Duchenne muscular dystrophy (DMD), demonstrates the formulation's unprecedented therapeutic potential, with up to 40% dystrophin expression restoration and 75% recovery of normal fiber functions, surpassing existing treatments and exhibiting substantial long‐term safety. This study presents a potent oral dosage form that dramatically improves UAA incorporation into target proteins in vivo, offering a significant advance in the treatment of nonsense mutation‐mediated disorders and holding considerable promise for clinical translation.

## Graphical abstract

This diagram illustrates the strategic approach of administering an oral formulation of an unnatural amino acid (UAA)‐based ionic liquids (ILs) to enhance dystrophin restoration in *mdx* mice through the MmPylRS‐tRNA_UUA_ system. The schematic delineates the pathway from oral delivery to cellular incorporation, highlighting the pivotal role of the UAA‐based IL in facilitating the targeted genetic code expansion for therapeutic intervention.

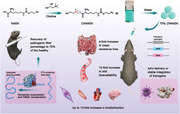

## 1 Introduction

Genetic code expansion (GCE) is a revolutionary technique that recodes premature termination codons (PTCs) into unnatural amino acids (UAAs), with significant applications spanning protein engineering,^[^ ^1^ ^]^ cellular process regulation,^[^ ^2^ ^]^ and heritable disease treatment.^[^ ^3^ ^]^ The development of orthogonal aminoacyl‐tRNA synthetase (aaRS)‐tRNA pairs has facilitated the specific incorporation of over 150 genetically encoded UAAs across various organisms.^[^ ^4^, ^5^, ^6^ ^]^ Our previous work demonstrated the potential of optimized PylRS–tRNA^Pyl^ pairs in addressing nonsense mutations within the Duchenne muscular dystrophy (DMD) gene and alleviating disease symptoms through UAA administration.^[^ ^3^, ^7^ ^]^ Despite these advancements, the clinical applicability of GCE is hampered by the low efficiency of UAA incorporation.^[^ ^8^, ^9^ ^]^

Efforts to enhance incorporation efficiency have focused on aaRS–tRNA pair evolution,^[^ ^10^ ^]^ translation machinery refinements,^[^ ^11^ ^]^ and orthogonal ribosome construction.^[^ ^12^ ^]^ However, these approaches necessitate complex cellular reprogramming and face limitations in in vivo applications.^[^ ^6^ ^]^ Furthermore, the in vivo delivery of hydrophobic UAAs remains a formidable challenge due to their poor solubility and formulation difficulties.^[^ ^13^, ^14^ ^]^ The rapid metabolism and the consequent requirement for high and frequent dosages of UAAs exacerbate the cost and complexity of treatments.^[^ ^15^, ^16^, ^17^ ^]^

Ionic liquids (ILs) and deep eutectic solvents, known for their high tunability and ease of preparation, present a promising solution.^[^ ^18^, ^19^ ^]^ Their capacity to solubilize, enhance permeation, and promote drugs across biomembranes is particularly noteworthy.^[^ ^14^, ^20^, ^21^ ^]^ This study harnesses the potential of active pharmaceutical ingredient (API)‐based ILs (API‑ILs), which can overcome the physicochemical limitations of drugs and physiological barriers, to optimize UAA delivery.^[^ ^14^ ^]^

**Figure 1:**
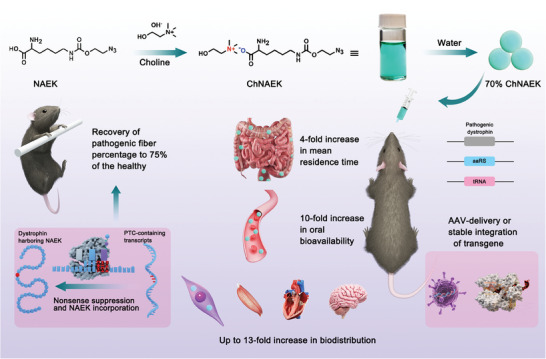
Conceptual Framework of the Study. This diagram illustrates the strategic approach of administering an oral formulation of UAA‐based ionic liquids (ILs) to enhance dystrophin restoration in *mdx* mice through the MmPylRS‐tRNA_UUA_ system. The schematic delineates the pathway from oral delivery to cellular incorporation, highlighting the pivotal role of the UAA‐IL complex in facilitating the targeted GC for therapeutic intervention. “UAA” denotes unnatural amino acids, and “IL” represents ionic liquids, both of which are integral to the novel therapeutic strategy depicted.

This study aims to tackle the low utilization efficiency of UAAs in GCE‐based research by utilizing three UAAs corresponding to well‐established GCE systems: NAEK (Nε ‐(2‐azidooxethyl) carbonyl)‐L‐lysine (hydrophilic), pAcF (L‐N‐acetylphenylalanine, hydrophobic), and Anap (3‐(6‐acetylnaphthalen‐2‐ylamino)−2‐aminopropanoic acid, hydrophobic).^[^ ^22^, ^23^, ^24^ ^]^ These UAAs were used as acidic APIs and paired with choline to create API‐ILs named ChNAEK, ChpAcF, and ChAnap. The study's findings reveal that our orally administered liquid formulation, based on UAA‐converted ILs, markedly increases the exposure and utilization of UAAs in vivo. Notably, there was a tenfold increase in oral bioavailability and up to a 13‐fold rise in lesion tissue accumulation. This led to robust dystrophin expression in mice, demonstrating the formulation's potential to recode PTCs within DMD transcripts efficiently (**Figure** **1**).

## 2 Results and Discussion

### 2.1 A Facile Oral Liquid Formulation Based on Ionic Liquids

**Figure 2:**
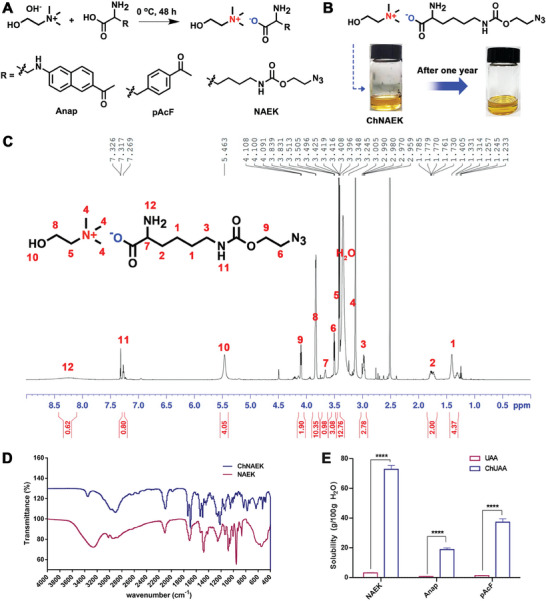
Synthesis and characterization of UAA‐based ILs. A) Illustrates the synthetic route for three distinct UAA‐based ILs, each entailing a specific UAA paired with choline to form a stable ionic liquid (IL). B) Displays the molecular structure of ChNAEK, alongside comparative visualizations of the substance prior to and following a stability assessment over one year, delineating the robustness of the IL's physical properties. Here, NAEK serves as the anion, and choline acts as the cation in the IL structure. C) Presents the ^1^H NMR spectra of ChNAEK in DMSO‐d6, providing detailed resonance assignments and coupling constants, indicative of the molecular integrity and composition of the IL. ^1^H NMR (600 MHz, DMSO‐d6): δ 8.26 (s, 2H), 5.46 (s, 4H), 4.10 (dd, J = 6.3, 3.8 Hz, 2H), 3.83 (dq, J = 5.4, 2.6 Hz, 8H), 3.52–3.49 (m, 2H), 3.43–3.41 (m, 9H), 3.13 (s, 39H), 2.99 (dd, J = 14.0, 7.6 Hz, 2H), 1.84–1.68 (m, 2H), 1.46 – 1.28 (m, 4H). D) Features the Fourier Transform Infrared (FTIR) spectra of ChNAEK and NAEK powder, with emphasis on the characteristic peak at 2900 cm^−1^, corresponding to the v(O–H) stretching in choline, signifying successful IL formation. E) Compares the solubility profiles of three UAAs and their respective API‐ILs in water, displaying a substantial solubility enhancement in the IL form. The data, represented as mean ± S.D. for *n* = 3, was subjected to rigorous statistical analysis using GraphPad Prism Software (Version 8.0, GraphPad Software, San Diego, CA), and the significance was determined through an unpaired two‐tailed Student's *t*‐test, with ^****^ *p* < 0.0001. This comprehensive characterization underscores the structural fidelity, stability, and enhanced solubility of the UAA‐based ILs, affirming their potential for therapeutic applications. Abbreviations: UAA, unnatural amino acids; IL, ionic liquids;NMR, nuclear magnetic resonance; API‐IL, active pharmaceutical ingredient‐ionic liquid.

To efficiently deliver UAAs orally and improve their in vivo utilization, we developed a liquid formulation utilizing a UAA‐based IL. Utilizing both hydrophilic and hydrophobic UAAs commonly employed in GCE, we synthesized ILs (ChNAEK, ChpAcF, and ChAnap) through neutralization reactions, following protocols established in prior research (**Figure** **2**A).^[^ ^25^ ^]^ Specifically, ChNAEK was produced by pairing NAEK(confirmed by ^1^H nuclear magnetic resonance (NMR) spectroscopy, Figure S1, Supporting Information) with choline in a stoichiometric ratio, resulting in a light‐yellow, transparent, and fluid liquid at ambient conditions, demonstrating over a year of stability (Figure 2B). The chemical identity and purity of ChNAEK were verified through mass spectrometry (Figure S2, Supporting Information), ^1^H NMR spectroscopy (Figure 2C), and Fourier Transform Infrared (FTIR) spectroscopy (Figure 2D), corroborating its structural integrity and composition.^[^ ^13^, ^26^ ^]^ Similarly, ChpAcF (Figure S3, Supporting Information) and ChAnap (Figures S3 and S4, Supporting Information) were synthesized and characterized, affirming successful formulation. Notably, the API‐IL form significantly enhanced the solubility of both hydrophobic and hydrophilic UAAs by over 20 times, with ChNAEK exhibiting a remarkable solubility exceeding 70% (m/v) in water (Figure 2E). This marked increase in solubility demonstrates the potential of API‐ILs to simplify UAA formulations and expand their biological applicability, indicating a promising avenue for the enhanced delivery of UAAs in genetic code expansion therapies.

To scrutinize the in vivo dynamics of UAA‐based ILs, we procured C57BL/6 mice (6–8 weeks old) from Vital River Laboratories and accommodated them under strictly regulated conditions (20–25 °C temperature, 40–70% humidity, and controlled lighting from 08:00–20:00) with unrestricted access to food and water. The comprehensive care and experimental protocols adhered rigorously to ethical guidelines and received full approval from the Committee of Animal Care and Use Institutions of Peking University (Approval No: LA2022354), ensuring adherence to the highest standards of animal welfare and research integrity.

**Figure 3:**
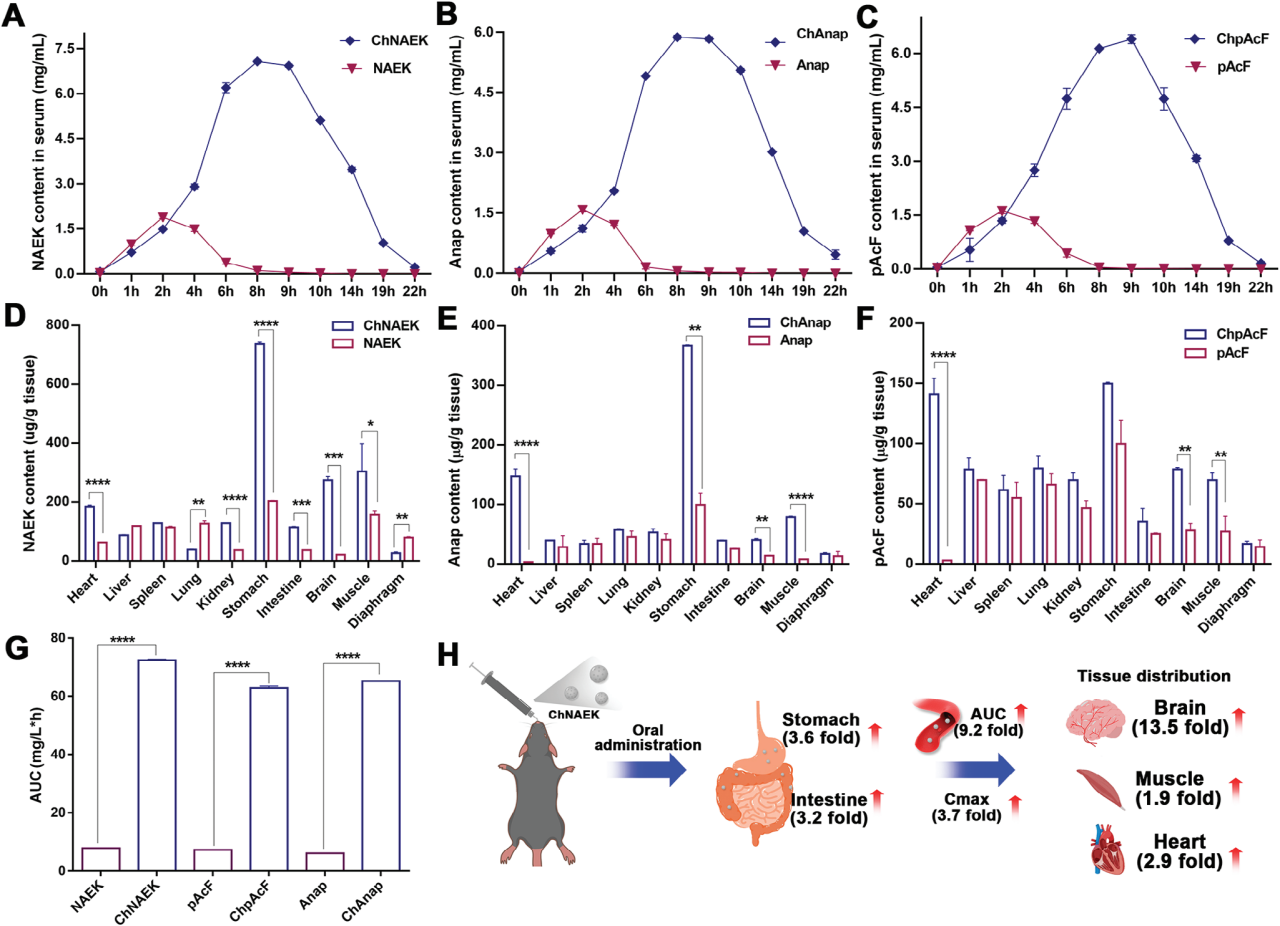
In vivo behavior of UAA‐based ILs and UAAs in wild‐type C57BL/6 mice (WT). A–C) Depict the pharmacokinetic profiles of three UAAs—NAEK, Anap, and pAcF—following oral administration of equivalent molar doses of either the free UAA or its corresponding IL form (ChNAEK, ChAnap, and ChpAcF, respectively), with each graph illustrating the temporal serum concentration curves of the UAAs in the mice (*n* = 3). D–F) Illustrate the in vivo tissue distribution of NAEK, Anap, and pAcF at 9.0 h post‐administration, detailing the relative concentrations of each UAA in major organs (*n* = 3). Notably, the IL forms (ChNAEK, ChAnap, and ChpAcF) exhibited markedly enhanced distribution in vital organs such as the stomach, heart, muscle, and brain compared to their free UAA counterparts, suggesting improved biodistribution and potential therapeutic efficacy. G) Compares the total UAA exposure (AUC) over 72 h for the oral administration of UAA‐based ILs against free UAA suspensions (*n* = 3), demonstrating significantly increased bioavailability of the IL formulations. H) Presents a schematic summarizing the impact of oral ChNAEK on altering NAEK's pharmacokinetic parameters and biodistribution, highlighting the substantial improvements in systemic exposure and targeted organ distribution. The results were meticulously analyzed using GraphPad Prism Software (Version 8.0, GraphPad Software, San Diego, CA) and presented as mean ± S.D., with statistical significance determined via an unpaired two‐tailed Student's *t*‐test (^*^ *p* < 0.05; ^**^ *p* < 0.01; ^***^ *p* < 0.001; ^****^ *p* < 0.0001). The comprehensive data delineates the enhanced pharmacokinetics and biodistribution profiles of UAA‐based ILs compared to free UAAs, underscoring their potential to optimize the delivery and therapeutic impact of UAAs in vivo.

Facile aqueous formulations of 70% (m/V) ChNAEK, 30% ChpAcF, and 15% ChAnap were meticulously prepared and administered orally via gavage to wild‐type (WT) C57BL/6 mice, with corresponding UAA solutions (50, 30, and 25 mg) serving as controls. We established a series of concentration gradients based on the viscosity, hydrophilicity, and gavage volume of the three UAA‐based ILs, ultimately optimizing the formulations for maximal survival and appropriate dosage volume in mice. Our pharmacokinetic analysis revealed that all API‐IL formulations significantly enhanced the pharmacokinetic profiles of UAAs compared to controls, evidenced by up to a tenfold increase in total UAA exposure (Area Under Curve, AUC) and higher serum concentrations of UAAs (**Figure** **3**A–C,G). Notably, the mean residence time (MRT) (e.g., from 3.30 to 9.72 h for NAEK) and Tmax (time to reach maximum serum concentration) (e.g., from 2 to 8 h for NAEK) were considerably extended (Table S1, Supporting Information), indicating prolonged exposure and sustained absorption of UAAs in vivo. The plasma Cmax (maximum serum concentration) of NAEK notably increased in the ChNAEK group compared to the control, with a marked extension in complete metabolism time (from 6 to 15 h) (Figure 3A). Specifically, the Cmax of NAEK was ≈1.89 mg mL^−1^ at 2 h after oral administration in the control group, while it reached ≈7.07 mg mL^−1^ at 8 h in the ChNAEK group. Similar enhancements in pharmacokinetic parameters were observed for ChAnap and ChpAcF formulations (Figure 3B,C; Table S1, Supporting Information). These findings underscore the capacity of UAA‐based ILs to significantly prolong UAA exposure times and improve their oral bioavailability, setting a precedent for enhanced utilization rates.

Additionally, the formulations influenced the biodistribution of UAAs, augmenting their presence in critical organs such as the muscle, brain, and heart, thereby providing targeted therapeutic potential (Figure 3D–F). The IL formulation notably increased NAEK concentrations in the gastrointestinal tract (over threefold), suggesting extended retention and improved absorption. Crucially, the amplified presence of NAEK in the brain (up to 13.5‐fold), muscle (1.94‐fold), and heart (2.95‐fold) in the ChNAEK group underlines the potential for the IL formulation to effectively deliver UAAs to deficiency‐affected tissues, offering a promising avenue for the treatment of related disorders. Enhanced tissue concentrations were similarly observed for the ChAnap and ChpAcF groups (Figure 3E,F), affirming the broad applicability and therapeutic promise of these UAA‐based IL formulations.

### 2.2 Suppression Efficiency of Ochre Stop Codon In Vitro and In Vivo

**Figure 4:**
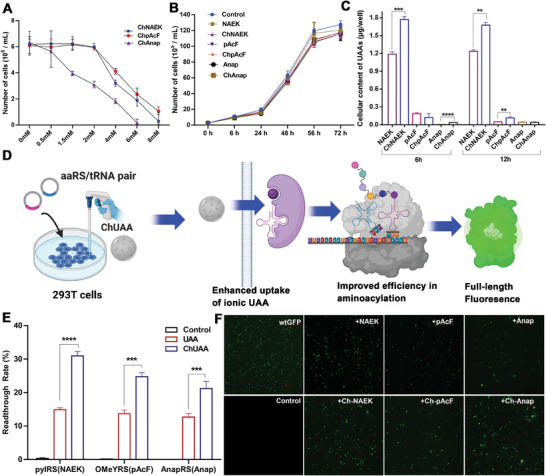
The readthrough efficiency of the ochre stop codon evaluated with the three UAA‐based ILs in mammalian cells. A) Quantifies the viability of 293T cells cultured in Dulbecco's Modified Eagle Medium (DMEM) containing varying concentrations of ChNAEK, ChpAcF, and ChAnap, providing insights into the cytocompatibility of these ILs (*n* = 3). B) Depicts the growth kinetics of 293T cells over 72 h in DMEM supplemented with 1 mm of either NAEK/pAcF or their corresponding ILs (ChNAEK/ChpAcF) and 100 µm of Anap or ChAnap, confirming no detrimental impact on cell proliferation under these conditions (*n* = 3). C) Illustrates the intracellular retention of UAAs in 293T cells post 6 and 12‐h incubations with equivalent molar concentrations of either UAAs or UAA‐based ILs, highlighting the enhanced cellular uptake of the latter (*n* = 3). D) Schematic elucidating the potential mechanisms through which UAA‐based ILs augment the restoration of GFP^39TAA^ in 293T cells. Following co‐transfection with suppression systems and GFP^39TAA^ plasmids, cells were treated with UAA‐based ILs (1 mm/100 µm) or free UAAs (1 mm/100 µm) for 48 h before being analyzed via fluorescence microscopy and flow cytometry to assess the readthrough efficiency of the mutated ochre codon. E) Compares the GFP^39TAA^ readthrough rates in 293T cells co‐transfected with suppression systems and GFP^39TAA^ plasmids, cultured in DMEM with either UAAs or UAA‐based ILs, as measured by cell fluorescence flow analysis (*n* = 3). F) Showcases GFP fluorescence images of 293T cells post co‐transfection with the suppression systems and GFP^39TAA^ plasmids in DMEM supplemented with UAAs or their respective ILs (*n* = 3). The data, presented as mean ± S.D., was statistically analyzed using an unpaired two‐tailed Student's *t*‐test, with significant differences noted at ^**^ *p* < 0.01; ^***^ *p* < 0.001; ^****^ *p* < 0.0001. These comprehensive analyses demonstrate the superior performance of UAA‐based ILs in promoting efficient stop codon readthrough, thereby potentiating their application in therapeutic strategies aimed at restoring full‐length proteins in diseases caused by premature stop codons.

Subsequent to establishing the enhanced pharmacokinetic profiles of UAA‐based ILs, we investigated their potential to facilitate the suppression of ochre stop codons through efficient recoding in disease‐relevant transcripts during protein translation, which has profound therapeutic implications. Initially, the cellular safety of ChNAEK, ChpAcF, and ChAnap was assessed in 293T cells to determine non‐toxic concentrations for further experiments.^[^ ^27^ ^]^ Optimal concentrations of 1 mm for ChNAEK/ChpAcF and 100 µm for ChAnap (**Figure** **4**B) were identified, with the maximum safe concentrations being 2 mM and 500 µm, respectively (Figure 4A). We then introduced three orthogonal aaRS‐tRNA pairs (pylRS‐tRNA^Pyl^_UUA_/OMeYRS‐tRNA^Tyr^_UUA_/AnapRS‐tRNA^Leu^_UUA_) into 293T cells alongside a green fluorescent protein harboring a premature termination codon (GFP^39TAA^) to evaluate UAA incorporation into the reporter protein. The results demonstrated that all three UAAs, when formulated as ILs, effectively participated in protein translation, with a markedly higher recoding efficiency of the mutated ochre codon compared to free UAA groups or untreated controls (Figure 4E,F; Figure S5, Supporting Information). Notably, the ChNAEK group exhibited the most substantial increase in incorporation efficiency, approximately doubling that of the NAEK group, while the ChAnap and ChpAcF groups also showed significant improvements (Figure 4E). The intracellular retention of UAAs from the API‐IL group was significantly higher (Figure 4C), laying the groundwork for enhanced UAA utilization in protein incorporation. This increase in readthrough efficiency of the PTC may be attributed not only to the intracellular retention of UAAs but also to their enhanced participation in tRNA aminoacylation. Research suggests that special UAAs are incorporated into proteins through a mechanism akin to tRNA amino acid ligation.^[^ ^28^, ^29^ ^]^ Given that ILs are known to catalyze esterification reactions, it's plausible that UAA‐based ILs might catalyze aminoacyl‐transfer reactions, esterifying UAAs to tRNA's 3′ terminus.^[^ ^30^, ^31^ ^]^ Thus, UAA‐based ILs may act as substrates with increased intracellular retention while also playing an autocatalytic role in UAA‐tRNA linkage mechanisms. This dual functionality could result in an elevated aminoacylation efficiency and, consequently, a significant enhancement in full‐length protein restoration (Figure 4D).

**Figure 5:**
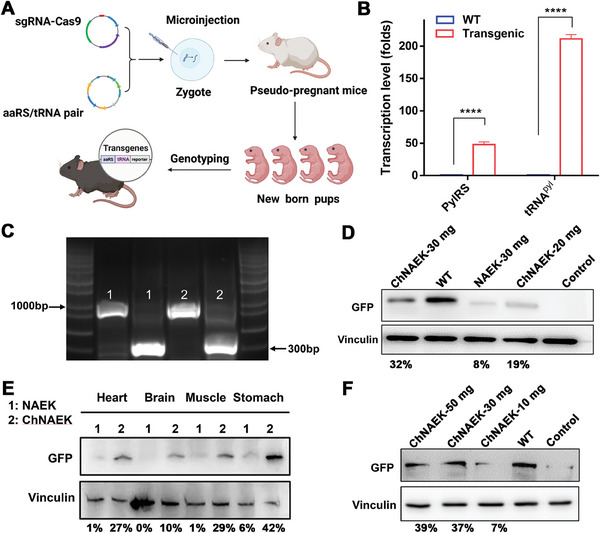
Efficiency of NAEK incorporation evaluated apparently in the heart, brain, muscle, and stomach tissues of transgenic mice. A) Illustrates the strategic methodology employed for engineering three distinct types of transgenic mice, each harboring an orthogonal aaRS/tRNA pair gene, laying the groundwork for specific UAA incorporation. B) Presents a comparative analysis of pylRS and tRNA^pyl^ transcription levels between the transgenic and WT mice, offering quantitative insights into the genetic modification's effectiveness (*n* = 3). Data are expressed as mean ± S.D., with statistical significance determined by an unpaired two‐tailed Student's *t*‐test (^****^ *p* < 0.0001). C) Depicts PCR analysis of tail DNA from successive generations of the transgenic mice, utilizing two specific primers (Primer 1 targeting a sequence at 900 bp and Primer 2 targeting a sequence at 300 bp) to confirm the presence and inheritance of the modified genes (*n* = 3), thereby verifying the successful establishment of the transgenic lines. D) Demonstrates the efficacy of NAEK incorporation through Western blot analysis of GFP restoration in transgenic mice post one‐week daily intramuscular injections of ChNAEK (20 and 30 mg) and NAEK aqueous solution (30 mg) (*n* = 2). E) Evaluates GFP restoration in the heart, brain, muscle, and stomach tissues of PylRS‐tRNA‐GFP39^TAA^ transgenic mice via Western blot, following 1 week of oral administration of equivalent molar doses of ChNAEK or NAEK solution (30 mg) (*n* = 2). F) Provides Western blot analysis of GFP restoration in transgenic mice post 1‐week oral administration of various doses (10, 30, and 50 mg) of ChNAEK (*n* = 2), illustrating the dose‐dependent efficacy of the oral formulation. The integrated optical density (IOD) of GFP bands from panels D, E, and F, normalized to wild‐type GFP levels and corrected for sample loading, quantitatively underscores the superior restoration capabilities of ChNAEK. Normalized IOD values are provided beneath each Western blot. These comprehensive analyses collectively confirm the enhanced efficiency of NAEK incorporation in vital organs of transgenic mice through the UAA‐based IL formulation, highlighting its potential in precision therapeutic applications.

To ascertain the in vivo efficiency of UAA incorporation into target proteins, we engineered three types of transgenic mice via prokaryotic microinjection, using plasmids to introduce genes conducive for UAA incorporation (**Figure** **5**A; Figure S6A, Supporting Information). Transgenic models designed for NAEK incorporation via the PylRS‐(U6‐ tRNA^Pyl^_UUA_)_4_‐GFP^39TAA^ gene exhibited marked transcription levels of aaRS and tRNA (Figure 5B; Figure S6B, Supporting Information),^[^ ^3^ ^]^ as confirmed by PCR analysis of mouse tail DNA (Figure 5C; Figure S7A,B, Supporting Information). Subsequent Western blot analysis, supplemented by gray value quantification, assessed the restoration of fluorescent protein GFP expression in these transgenic mice. The study revealed a notable increase in GFP expression following a one‐week daily intramuscular injection regimen of ChNAEK compared to an equivalent molar dose of free NAEK. Notably, a lower dose of ChNAEK (equivalent to 20 mg of NAEK) achieved a higher incorporation rate (19%) than a higher dose of free NAEK (30 mg, 8%) (Figure 5D). Similar trends were observed for ChpAcF and ChAnap in respective transgenic mouse models, indicating that UAA‐based ILs markedly improve the in vivo utilization rate of UAAs forGCE incorporation. Further exploration revealed enhanced GFP expression in the heart, brain, stomach, and muscle tissues of transgenic mice administered ChNAEK orally, surpassing the results from the NAEK aqueous solution group (Figure 5E). This enhancement aligns with the observed increased tissue accumulation and distribution of NAEK, particularly notable in the stomach, which displayed the highest GFP restoration post‐oral administration. Analogous outcomes were obtained for pAcF (Figure S9A, Supporting Information) and Anap (Figure S9B, Supporting Information) following oral administration, demonstrating the benefits of enhanced UAA content in focal tissues post‐oral administration. These findings highlight the synergistic effect of tuned biodistribution and enhanced UAA utilization in the IL groups.

Optimization of the oral dose for UAA incorporation revealed that 30 mg of ChNAEK was the most effective, as higher doses did not significantly increase GFP expression (Figure 5F). Similar dosage optimizations and findings were observed with ChpAcF (Figure S10, Supporting Information), suggesting a saturation point in protein restoration at certain doses. Interestingly, oral administration of 30 mg ChNAEK yielded comparable GFP restoration (37%) to that achieved by intramuscular injection (32%), suggesting a potential dosage ceiling for protein read‐through in muscle cells.

This comprehensive analysis underscores the profound impact of UAA‐based IL formulations in enhancing the efficacy of genetic code expansion techniques in ***vivo***, paving the way for improved therapeutic strategies for disorders necessitating targeted protein restoration.

### 2.3 Dystrophin Restoration Efficiency in *mdx* Mice

To elucidate the therapeutic potential of UAA‐based ILs for treating nonsense mutation‐induced diseases, we focused on DMD, a condition precipitated by a nonsense mutation in exon 23 of the dystrophin gene. Using the C57BL/10ScSn‐*Dmd* ^mdx^/Jmouse strain（ chrX:g.83803333 C>T; p.Q995*) from Jackson laboratory, we conducted experiments with the approval of Peking University's Committee of Animal Care and Use Institutions. GCE offers a compelling alternative to gene therapy for DMD, aiming to restore full‐length dystrophin via protein translation rather than relying on mini‐ or micro‐dystrophin constructs.^[^ ^32^ ^]^ This approach avoids the potential safety concerns associated with off‐target mutations in CRISPR‐based genome editing and the undesired effects of antisense oligonucleotide‐based therapy.^[^ ^33^ ^]^ However, the historically low in vivo utilization efficiency of UAAs has been a significant bottleneck.

**Figure 6:**
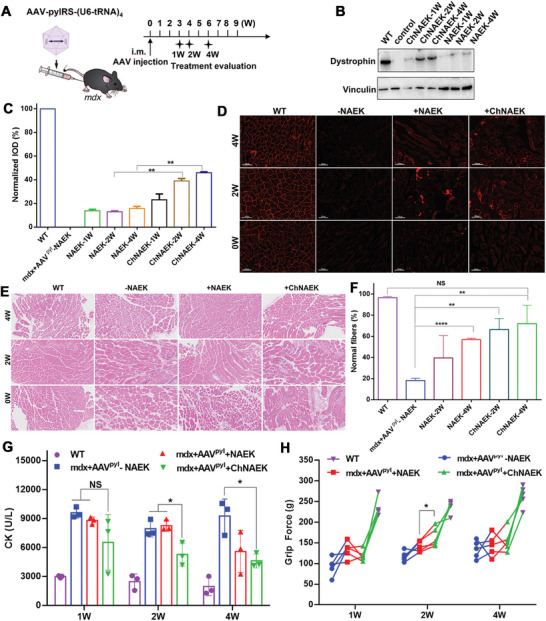
Oral delivery of ChNAEK improved the efficiency of dystrophin restoration by up to 40% in 4 weeks. A) Provides a detailed schematic of the AAV‐PylRS‐(U6‐tRNA_UUA_)_4_ vector construction and its intramuscular (IM) injection into *mdx* mice, outlining the subsequent oral treatment regimen and the timeline for therapeutic efficacy evaluation at 1, 2, and 4 weeks post‐administration. B) Shows Western blot analysis of dystrophin restoration in the tibialis anterior muscle of *mdx* mice post‐AAV IM injection, with daily oral administration of either NAEK or ChNAEK (30 mg), over the course of 1, 2, and 4 weeks (*n* = 3). C) Depicts the integrated optical density (IOD) analysis of dystrophin restoration over the same time periods, with dystrophin band intensities normalized against those of the WT mice group (*n* = 2). The data are presented as mean ± S.D., and statistical significance assessed via an unpaired two‐tailed Student's *t*‐test (^**^ *p* < 0.01). D) Illustrates dystrophin immunofluorescence in tibialis anterior muscles from *mdx* mice treated for 0, 2, and 4 weeks, showing marked red fluorescence indicative of dystrophin restoration in the NAEK or ChNAEK treated groups (*n* = 3). E) Presents Hematoxylin and Eosin (H&E) staining of tibialis anterior muscles from the same sets of mice, providing histological insights into the muscle's condition post‐treatment (*n* = 3). F) Quantifies the percentage of normal fibers by counting myofibers with peripherally located myonuclei in H&E‐stained sections, with five regions analyzed per group (*n* = 3). Data are expressed as mean ± S.D., with statistical analysis conducted using an unpaired two‐tailed Student's *t*‐test (^**^ *p* < 0.01; ^****^ *p* < 0.0001; NS, non‐significant). G) Monitors serum creatine kinase (CK) concentrations as a diagnostic marker for muscle disease across different time points post‐treatment (*n* = 3), with results presented as mean ± S.D. and analyzed via an unpaired two‐tailed Student's *t*‐test (^*^ *p* < 0.05, NS, non‐significant). H) Assesses the grip strength of *mdx* mice post‐treatment to evaluate muscle function, with muscle strength of each limb measured using a grip strength meter (*n* = 5). The statistical analysis was performed using a two‐sided one‐way analysis of variance with Tukey's post hoc test (^*^ *p* < 0.05). This comprehensive dataset unequivocally demonstrates the superior efficacy of oral ChNAEK treatment in promoting dystrophin restoration, muscle fiber normalization, and functional recovery in *mdx* mice, positioning it as a highly promising therapeutic modality for DMD treatment.

Given our preceding findings, we hypothesized that UAA‐based ILs could significantly improve the bioavailability and distribution of UAAs, particularly in critical tissues like muscles, heart, and brain, where dystrophin deficiency can exacerbate disease progression and lead to fatal outcomes in DMD patients.^[^ ^34^ ^]^ To test this hypothesis, *mdx* mice injected with AAV‐PylRS‐(U6‐tRNA_UUA_)_4_ were orally administered with either a NAEK solution or equimolar doses of the ChNAEK formulation (**Figure** **6**A). The results were promising: the ChNAEK group showed a threefold increase in full‐length dystrophin restoration compared to the free NAEK group after 2 weeks of treatment (Figure 6B,C). This result significantly surpassed the restoration achieved by free NAEK in a previous intraperitoneal injection study.^[^ ^3^ ^]^ Moreover, no substantial difference in dystrophin restoration was observed between the 2‐ and 4‐week ChNAEK treatment groups, with both achieving ≈40% restoration, suggesting a plateau in protein restoration within this timeframe. This finding indicates that a shorter period and lower total UAA dose are required to reach the restoration plateau compared to previous methods. Additionally, dystrophin restoration in the ChNAEK group was also elevated after just one week of either intramuscular or intraperitoneal injection in *mdx* mice (Figure S11A,B, Supporting Information). These results affirm the enhanced therapeutic potential of UAA‐based ILs in treating DMD by efficiently restoring full‐length dystrophin, offering a promising avenue for more effective and potentially faster treatment strategies for this and similar genetic disorders. Immunofluorescence analysis of muscle tissues revealed a substantial increase in full‐length dystrophin expression following 2 and 4 weeks of oral ChNAEK treatment (Figure 6D). In contrast, the control group without NAEK treatment displayed no specific fluorescence, indicating an absence of full‐length protein recovery. Notably, the red fluorescence intensity, indicative of dystrophin presence, was significantly greater in the ChNAEK 4‐week group than in the free NAEK group.

Muscle recovery in *mdx* mice was further substantiated by histological evaluations using hematoxylin‐eosin (H&E) staining. Both the 2‐ and 4‐week ChNAEK treatment groups exhibited substantial morphological improvements in muscle tissues compared to the non‐treated group. These improvements included a marked reduction in inflammatory lesions, an increase in the proportion of normal muscle cells, and a decrease in nuclear displacement (Figure 6E,F). Remarkably, the percentage of normal fibers—assessed by the location of myonuclei at the periphery of the fibers in H&E stained sections—improved significantly, reaching up to 75% of WT levels (Figure 6F). The therapeutic efficacy of ChNAEK was further corroborated by physiological measurements. Over the course of 1, 2, and 4 weeks of oral NAEK or ChNAEK administration, there was a significant and progressive reduction in serum creatine kinase (CK) levels toward normal values (Figure 6G), and a concomitant increase in the grip strength of the mice (Figure 6H). The improvements observed in the ChNAEK group, in both CK levels and grip strength, were markedly superior to those in the free NAEK group.

These findings collectively indicate that ChNAEK significantly enhances the bioavailability and utilization of NAEK in *mdx* mice, leading to efficient and substantial restoration of dystrophin expression and consequential improvements in muscle cell development and metabolism. This method not only outperforms previous treatment modalities in terms of recovery rate and therapeutic effectiveness but also offers a more efficient, durable, and convenient approach for the oral treatment of DMD, requiring lower UAA doses and enabling faster, more effective treatment outcomes.

In Section 2.2, we undertook a rigorous comparative analysis of PTC readthrough efficacy between UAAs and UAA‐based ILs across cellular and transgenic mouse models. Our research delineated a marked enhancement in GFP readthrough rates when utilizing all three UAA‐based IL formulations. Notably, the ChNAEK group manifested a doubling in intracellular incorporation efficiency relative to the free NAEK group (Figure 4E). Furthermore, administering a 30 mg dose of ChNAEK resulted in a fourfold increase in GFP restoration compared to NAEK after one week of intramuscular administration (Figure 5D) and a 29‐fold increase following one week of oral administration (Figure 5E), with both administration routes yielding comparable levels of GFP or dystrophin expression. However, it's worth noting that while oral administration of ChNAEK demonstrated remarkable efficacy in protein restoration, it did not significantly enhance CK levels or grip strength in *mdx* mice over NAEK after the initial week. A distinct improvement in these metrics was observed after 2 weeks. This lag may be attributed to the asynchronous nature of dystrophin restoration and functional recovery in *mdx* mice, suggesting that more time might be necessary to observe substantial improvements in dystrophin functionality. This insight points to the need for extended therapeutic regimes to fully realize the benefits of UAA‐based IL formulations in the treatment and functional recovery of DMD and potentially other genetic disorders.

### 2.4 In Vivo Safety Evaluation

**Figure 7:**
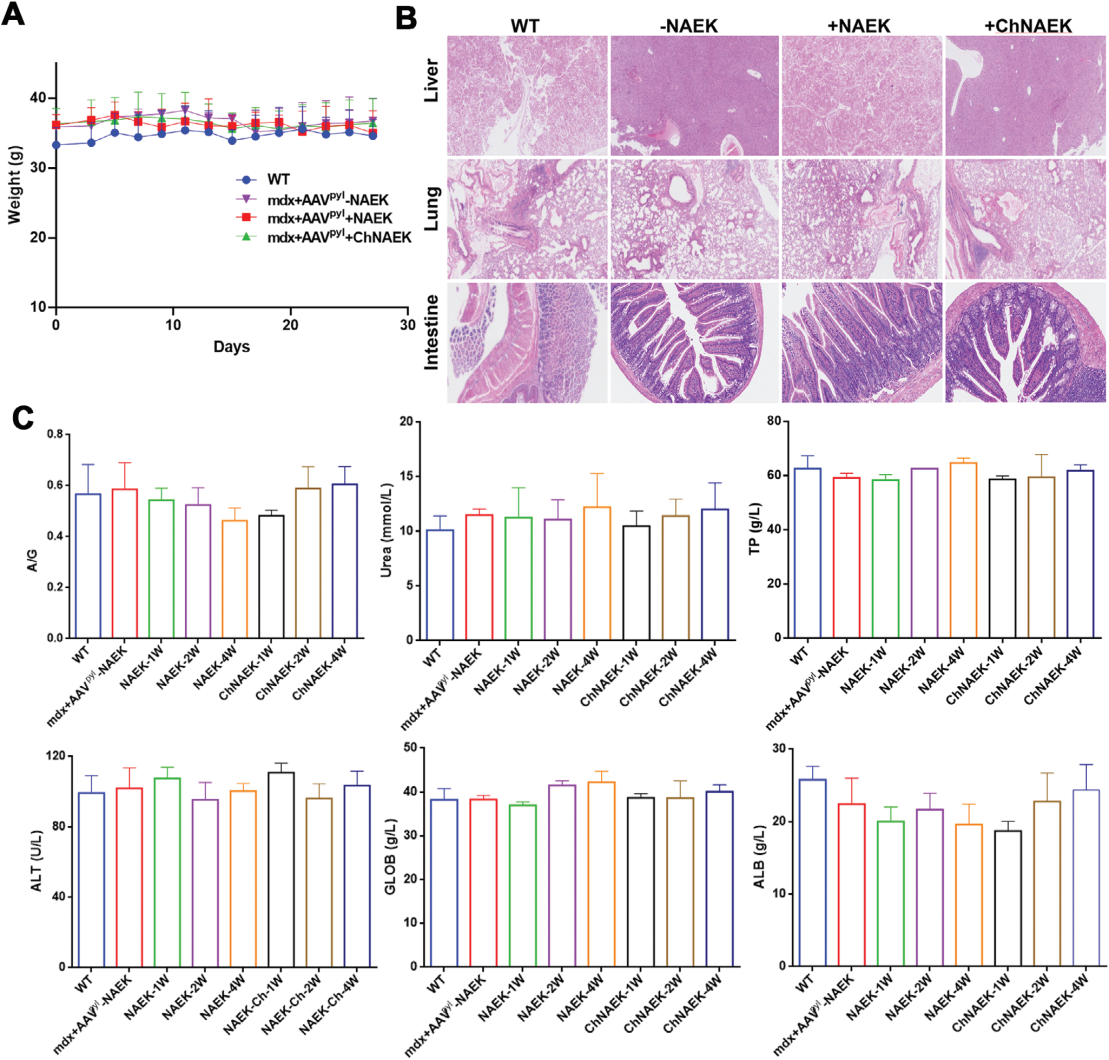
In vivo safety evaluation of the *mdx* mice treated with ChNAEK. A) Illustrates the body weight‐time curves over a 28‐day period for four distinct mouse groups, including those treated with ChNAEK, providing a longitudinal view of the treatment's impact on overall health and well‐being (*n* = 4). B) Showcases the toxicological assessment of the PylRS–tRNA^Pyl^ system and ChNAEK on dystrophin‐deficient tissues such as the liver, lung, and intestine at 4 weeks post‐treatment, utilizing H&E staining to discern any histopathological alterations (*n* = 3). C) Depicts a comprehensive serum biochemical analysis comparing the levels of ALB, GLOB, A/G, urea,TP, and ALT in *mdx* mice treated with ChNAEK for 1, 2, and 4 weeks, against WT mice, NAEK aqueous solution‐treated mice, and *mdx* mice without NAEK treatment (*n* = 3). The data are expressed as mean ± S.D., with statistical significance assessed using one‐way ANOVA followed by Tukey multiple comparisons tests, revealing no significant differences and thereby affirming the safety profile of ChNAEK treatment. This comprehensive evaluation elucidates the non‐toxic nature of ChNAEK in *mdx* mice, confirming its safety across multiple physiological and biochemical parameters over an extended period.

In our quest to ascertain the in vivo safety of the oral liquid ChNAEK formulation, we conducted a comprehensive assessment in WT C57BL/6 mice. Observational data including body weight and survival curves (**Figure** **7**A; Figure S12, Supporting Information) revealed no significant weight loss or mortality among *mdx* mice post‐AAV virus injection and subsequent ChNAEK administration, compared to the untreated control group. This suggests a favorable safety profile for ChNAEK at the administered doses. Further, histopathological examinations via Hematoxylin and Eosin (H&E) staining of liver, lung, and intestine tissues harvested after 4 weeks of oral ChNAEK treatment disclosed no discernible adverse effects on the mice's tissues and organs (Figure 7B). Systematic evaluations of different organ and tissue morphologies post one week of oral administration of either ChNAEK or NAEK solution (Figure S13, Supporting Information) corroborated these findings, indicating no significant pathological disparities between the experimental and WT groups. Biochemical analyses measuring parameters such as alanine aminotransferase, albumin (ALB), globulin (GLOB), the albumin‐globulin ratio (A/G), urea, total protein (TP), and glutamic pyruvic transaminase (ALT) were performed. The results demonstrated no notable alterations in these parameters in *mdx* mice after 1, 2, and 4 weeks of oral treatment with either free NAEK solution or an equivalent molar dose of ChNAEK (Figure 7C). This lack of significant variation suggests that moderate accumulation of ILs in organs does not induce adverse side effects. Interestingly, the accumulation of ChNAEK in organs such as muscles, the heart, and the brain is deemed beneficial for restoring normal dystrophin expression in these tissues, which is crucial for treating DMD. Furthermore, ionic liquids have shown promise in enhancing drug delivery to the brain, where their accumulation can be advantageous.^[^ ^35^ ^]^ Notably, the biological activity of choline, a component of the IL, is essential for various bodily functions, including reducing pathologies associated with Alzheimer's Disease when consumed in adequate amounts.^[^ ^36^ ^]^ Ionic liquids composed of choline and amino acids have been recognized as green, functional materials with biocompatibility.^[^ ^37^ ^]^ The absence of significant adverse effects underscores the potential of ChNAEK as a viable and safe therapeutic option for Duchenne muscular dystrophy and possibly other conditions necessitating UAA incorporation.

In the realm of therapeutic advancements for DMD caused by nonsense mutations, many existing strategies are hampered by low efficiency and the occurrence of side effects.^[^ ^38^ ^]^ Our study distinguishes itself by utilizing an oral UAA‐based IL formulation, specifically ChNAEK, which significantly outperforms traditional therapies. We achieved ≈40% restoration of dystrophin expression and up to 75% recovery of muscle fiber function in *mdx* mice, surpassing the results from free NAEK, aminoglycoside drugs like PTC124, and micro‐ or mini‐dystrophin delivery methods.^[^ ^39^, ^40^ ^]^ Notably, PTC124 and AAV‐CRISPR‐Cas9 system‐based treatments have shown considerably lower restoration rates in previous studies.^[^ ^41^ ^]^ The remarkable efficiency of ChNAEK is further underscored by its comparison with RNA base editing strategies, which have demonstrated substantially lower editing (2.4%) and dystrophin expression rates (only 2.5%).^[^ ^42^ ^]^ Importantly, our IL formulation achieved significant therapeutic effects with a lower total dose of UAA and without inducing noticeable side effects. This safety profile is particularly advantageous compared to CRISPR‐based therapies, which pose risks associated with genomic integrity and transcriptome equilibrium.^[^ ^41^, ^43^ ^]^

Our study's success builds on the broader potential of UAAs in therapeutic applications, as evidenced by the recent breakthroughs in antibody‐drug conjugates like ARX788, which incorporate UAAs for targeted treatment. The underlying mechanism of our approach, leveraging various orthogonal aaRS‐tRNA pairs, is versatile and can be adapted to manipulate protein function and treat a wide array of diseases caused by nonsense mutations. our oral ChNAEK‐based therapy represents a significant leap forward in the treatment of DMD and potentially other genetic disorders, offering a more efficient, safer, and patient‐friendly approach compared to existing therapies. As the field of UAA incorporation continues to evolve, our strategy opens new avenues for precision medicine, with the potential to revolutionize treatment paradigms for diseases caused by nonsense mutations.

### 2.5 Statistical Analysis

All experimental data were statistically analyzed using GraphPad Prism Software (Version 8.0, GraphPad Software, San Diego, CA) and presented as mean ± standard deviation (SD, *n* ≥ 3). A statistically significant difference was considered when the value of ^*^ *p* < 0.05 and very significant when *p* < 0.01 (^**^ *p* < 0.01; ^***^ *p* < 0.001; ^****^ *p* < 0.0001), using unpaired Student's *t*‐test (two‐tailed) for statistical analysis of two‐group comparison or one‐way ANOVA followed by Tukey multiple comparisons tests.

## 3 Conclusion

This study presents a novel and accessible oral liquid formulation utilizing UAA‐based ILs, designed to restore and modify endogenous proteins in vivo. Through extensive therapeutic efficacy and safety evaluations at the disease mouse model level, we've established the ChNAEK formulation as a groundbreaking advancement in the treatment of DMD via the MmpylRS‐tRNA_UUA_ system. The significant muscle function recovery and symptom mitigation observed in *mdx* mice underscore the unprecedented therapeutic potential of this approach. Our research contributes a viable and efficient oral formulation that significantly enhances the incorporation of UAAs into target proteins in vivo, marking a substantial leap forward in GCE therapies. The implications of this study extend beyond DMD, offering a versatile platform for the treatment of various diseases caused by nonsense mutations and potentially revolutionizing the field of molecular medicine with a focus on patient accessibility and improved quality of life.

## Conflict of Interest

The authors declare no conflict of interest.

## Supporting information

Supporting Information

## Data Availability

The data that support the findings of this study are available from the corresponding author upon reasonable request.
