# Supplementary material for: Unnatural Amino Acid‐Based Ionic Liquid Enables Oral Treatment of Nonsense Mutation Disease in Mice
Source: Adv Sci (Weinh). 2024 Jan 30;11(13):2306792. doi: 10.1002/advs.202306792 (PMC10987103; doi:10.1002/advs.202306792)
Supplement: Supplementary file 1 — Supporting Information [file ADVS-11-2306792-s001.pdf]

ADVANCED SCIENCE
Open Access

Supporting Information

for *Adv. Sci.*, DOI 10.1002/advs.202306792

Unnatural Amino Acid-Based Ionic Liquid Enables Oral Treatment of Nonsense Mutation Disease in Mice

Yujie Shi, Ningning Shi, Yuelin Yang, Zhetao Zheng and Qing Xia**

Supporting information

Unnatural amino acid-based ionic liquid enables oral treatment of nonsense mutation disease in mice

Yujie Shi [1,2,3*], *Ningning Shi*[1,4], *Yuelin Yang*[1,4], *Zhetao Zheng*[1,4], *Qing Xia*[1,4*]

Materials and methods

Synthesis and characterization of ChNAEK, ChAnap and ChpAcF

This section delineates the methodical approach employed for the synthesis and subsequent characterization of the ionic liquids (ILs) derived from unnatural amino acids (UAAs) namely NAEK, Anap, and pAcF, as per the methodology outlined in referenced literature. The specific synthesis pathway is illustrated in Figure 1A and Supplementary Figure S1.

For ChNAEK synthesis, an accurately measured quantity of NAEK was dissolved in an appropriate volume of water within a reaction vessel and subjected to agitation until fully dissolved. Following this, choline hydroxide, initially containing 45% methanol, was concentrated using a rotary evaporator. The concentrated choline hydroxide (in a 1:1 molar ratio to NAEK) was then incrementally added to the NAEK solution using a glass dropper. The reaction mixture was maintained at 0°C for 48 hours to facilitate reaction completion. Post-reaction, the product was concentrated at 55 ℃ using a rotary evaporator. Subsequently, the amino acid precipitant ACN/MeOH (9/1) was added, and the resultant mixture was filtered. The filtrate was then subjected to rotary evaporation at 40℃ to remove organic solvents, followed by drying in a vacuum oven at 60℃ for 48 hours to yield the final liquid product, ChNAEK. The synthesis protocol for ChpAcF followed a similar methodology. Due to Anap's pronounced hydrophobicity, the molar ratio of choline to Anap in ChAnap synthesis was adjusted to 6:1.

Upon successful synthesis of the three UAA-based ILs, a comprehensive characterization was performed. Mass spectrometry analyses of the precursor UAAs and resultant UAA-based ILs were conducted using an API4000 triple quadrupole series mass spectrometer with a Turbo Ionspray

ion source (AB SCIEX, USA). Infrared (IR) spectroscopic analysis was carried out using a FITR spectrometer (Thermo Fisher, USA) to elucidate the molecular vibrations and chemical bonds. Furthermore, ^{1}H NMR spectroscopy was performed on an AVANCE III 400MHz NMR spectrometer (Bruker, Switzerland) to confirm the chemical structure and purity of the UAA-based ILs.

Additionally, the aqueous solubility of the UAAs (NAEK, Anap, and pAcF) and the corresponding UAA-based ILs was determined by measuring the mass of each compound that reached saturation in 100 g of deionized water (ddH2O). This metric provided a quantitative assessment of the solubility enhancements achieved through IL formation.

Cell Culture and Transfection

HEK293T cells (ATCC CRL-11268) were cultured in Dulbecco's Modified Eagle Medium (DMEM, Corning) supplemented with 10% fetal bovine serum (FBS, Gibco) and maintained at 37°C in a humidified atmosphere containing 5% CO_2. For the readthrough assay, pCMV-EGFP vectors harboring a site-specific TAA nonsense mutation at position 39 were co-transfected with PylRS-tRNA^{Pyl}, OMeYRS-tRNA^{Tyr}, or AnapRS-tRNA^{Leu} plasmids into HEK293T cells. The transfection was facilitated using Megatran 2.0 reagent (Origene) following the manufacturer's protocol. Six hours post-transfection, the cell culture medium was replaced with fresh DMEM supplemented with NAEK (ChNAEK) /pAcF (ChpAcF) (1mM) or Anap (ChAnap) (100 µM). The cells were then incubated for an additional 48 hours to allow for sufficient expression and readthrough of the mutated GFP. Post-incubation, GFP fluorescence was assessed using a fluorescence microscope (Nikon) to qualitatively visualize the efficacy of the nonsense mutation suppression by the UAA-based ILs. Each treatment group was conducted in triplicate to ensure the reliability and reproducibility of the results.

Cell Viability Assay and Cell Growth Curve

To evaluate the impact of UAA-based ionic liquids (ILs) on cellular viability and proliferation, a series of preliminary preparations and dilutions were conducted. Initially, precise quantities of UAA-based ILs were weighed and subsequently dissolved in water to create three distinct 50 mM aqueous solutions of the UAA-based ILs. These solutions served as stock concentrations from

which further dilutions were made. Subsequently, the culture medium for 293T cells was supplemented with varying concentrations of the UAA-based ILs. A gradient of dilutions was prepared to encompass a comprehensive range of concentrations, including 0 mM (control), 0.5 mM, 1.5 mM, 2 mM, 4 mM, 6 mM, and 8 mM. These varying concentrations were meticulously prepared to assess the cellular response across a spectrum of IL exposure levels. The prepared media containing the specified concentrations of UAA-based ILs were then applied to 293T cell cultures. Following treatment, cell viability assays and growth curve analyses will be conducted at designated time points to quantitatively assess the cytotoxicity and proliferative effects of the UAA-based ILs.

Cell Planking

Cells from 10cm dishes were digested into single cells using 1 mL of 0.25% Trypsin-EDTA and resuspended, and the cell count was determined using a cell counter. The cells were then seeded into a six-well plate at a density of 3×10^5 cells per well and incubated overnight at 37°C. Upon reaching about 70% confluence, transfection was performed. Cells were treated with medium containing different concentrations of UAA-based ILs, observed for 48 hours for cell status and viability. After 48 hours, cells were digested, collected, and counted using a cell counter to create a fitting curve of cell number and UAA-based ILs concentration.

Cell Growth Curve

Two experimental groups were established, one with UAA aqueous solution and the other with an equal molar amount of UAA-based ILs added to 293T cells. Three replicate wells were cultured for each group. Cells from each group were collected and counted at 0 h, 6 h, 24 h, 48 h, 56 h, and 72 h to plot cell growth curves under different conditions.

Cellular Internalization of UAAs

293T cells were cultured in six-well plates at 4×10^5/well. Six groups were established, and three replicate wells of cells were used for each group. The medium was replaced with fresh DMEM containing NAEK/pAcF (1 mM), Anap (100 μM), Ch-NAEK/Ch-pAcF (1 mM), or Ch-Anap (100 μM). Cells were incubated for an additional 6 and 12 hours. Cells from each group were collected, washed with PBS three times, then resuspended in 100 μL dH2O for 5 minutes,

and centrifuged at 12000 g for 10 minutes. The UAA concentration in the supernatant from each group was quantified using high-performance liquid chromatography–mass spectrometry (HPLC-MS) (Waters ACQUITY UPLC H-Class, Waters Xevo TQ-S).

Flow Cytometry and FACS

To quantify the proportion of GFP-positive HEK293T cells indicative of successful readthrough of the targeted nonsense mutation, flow cytometry and fluorescence-activated cell sorting (FACS) were employed 48 hours post-medium change. GFP-positive HEK293T cells were collected 48 hours after medium change, digested into single cells using trypsin/EDTA, and washed three times with PBS. The cells were then transferred into 1.5 mL ep tubes containing 500 μL PBS and analyzed on a BD FACSAria (BD Biosciences) with appropriate filter settings (488nm-FITC coherent sapphire laser for GFP excitation). Data were analyzed using FlowJo software.

qRT-PCR Analysis

To quantitatively evaluate the gene expression levels in the tibialis anterior muscle tissues of both transgenic and wild-type (WT) mice, a rigorous qRT-PCR analysis was conducted. Total RNA from transgenic or WT mice's tibialis anterior muscle was extracted using the Quick-RNA MicroPrep kit (Zymo Research), reverse-transcribed, and then subjected to quantitative PCR with the LightCycler® System (Roche). Target gene transcripts were normalized to internal GAPDH control, and relative gene expression fold was calculated using the $2^{-\Delta\Delta Ct}$ method. qRT-PCR primer details are provided in Table S2.

Genotype Identification of Transgenic Mice by Tail DNA PCR Analysis

To ascertain the genotypes of the three distinct transgenic mouse lines, a comprehensive PCR analysis of tail DNA was conducted. Tail samples from young generation mice were collected into 1.5 mL centrifuge tubes on ice, and 500 μL of cracking solution (containing 50 μg protease) was added. The subsequent DNA extraction was meticulously performed following the standard protocol provided by the genomic DNA extraction kit (TIANGEN), ensuring the purity and quality of the isolated DNA. Post-extraction, the concentration of the obtained DNA was accurately measured to determine its suitability for PCR amplification. A precise quantity of 200 ng of DNA

from each sample was then used as the template for the PCR analysis. The PCR was conducted using specific primer sequences designed to target the unique genetic modifications of each transgenic mouse line. These primer sequences, integral to the accurate identification of the transgenic genotypes, are comprehensively listed in Supplementary Table S3.

Bioavailability and Tissue Distribution of UAAs from UAA Solutions/UAA-based ILs

In evaluating the bioavailability and tissue distribution of unnatural amino acids (UAAs) and their respective ionic liquid (IL) derivatives, wild-type C57BL/6 mice were orally administered with UAAs or equimolar doses of UAA-based ILs. Serum samples were collected at specified intervals (0 h to 22 h post-administration) for UAA quantification. The protein in the serum was precipitated using acetonitrile, and the UAA-containing supernatants were collected for analysis at different time points. Additionally, various tissues were harvested 9 hours post-administration for UAA quantification. High-performance liquid chromatography–mass spectrometry (HPLC-MS) (Waters ACQUITY UPLC H-Class, Waters Xevo TQ-S) was employed for UAA concentration analysis as per established protocols. Pharmacokinetic parameters were calculated using the DAS (Data Analysis System) software package (Version 2.0, BioGuider Co., Shanghai, China) according to a one-compartment model.

Western Blotting Analysis

Post one, two, or four weeks of oral administration of ChNAEK or NAEK, tissue samples from the mice were homogenized, and cellular debris was removed via centrifugation at 4°C. Proteins from different groups were extracted using lysis buffer for 10 minutes and then quantified using a BCA assay (Thermo). A total of 50 μg of protein from each sample was boiled with loading buffer, separated on 4-12% Nu-PAGE (Invitrogen), and transferred onto a polyvinylidene difluoride membrane overnight. The membrane was blocked with 5% (v/v) non-fat milk in TBST (50 mM Tris-HCl, 150mM NaCl, and 0.02% Tween-20, pH 7.5) at room temperature for 1 hour and then incubated with rabbit or mouse polyclonal antibodies overnight at 4°C. Antibodies used included anti-dystrophin (1:500, ab7164, Abcam), anti-vinculin (1:3,000, ab91459, Abcam), anti-GFP (1:3,000, 66002-1-Ig, Proteintech), and anti-mCherry (ab183628) diluted in TBST containing 5% (v/v) defatted milk. Membranes were washed three times with TBST, incubated with horseradish peroxidase-conjugated goat anti-rabbit/mouse IgG (1:3,000) at room temperature for

1 hour, developed using an enhanced chemiluminescent detection kit (Millipore), and visualized using an automated chemiluminescent image analysis system (Tanon). The integrated optical density (IOD) of western blot bands was semi-quantified using the ImagePro Plus software package.

AAV Construction and Administration

The AAV- pylRS-tRNA$^{Pyl}_{UUA}$ construct comprised four tRNA copies under a U6 promoter and a full-length PylRS with a Myc tag under the CMV promoter at the C-terminal. As a control, the AAV-CMV-mScarlet-3-Myc flag excluded the PylRS-tRNA. Both were packaged into the AAV2/9 serotype, with the AAV-PylRS-tRNA vector having a titre of 4.10×10^{13} viral genomes (vg)/mL. Male *mdx* mice, aged 6–8 weeks, were anesthetized for the topical administration of 50 μL each of AAV-PylRS-tRNAPyl and control AAV into their right and left tibialis anterior muscles, respectively. Daily oral administration of either NAEK (30 mg) or an equimolar dose of ChNAEK followed. At 1, 2, and 4 weeks post-administration, some mice were euthanized for tissue and blood analyses. The body weight and survival rates were monitored and recorded.

Immunofluorescence Staining

Tibialis anterior muscles from various groups were fixed in 4% paraformaldehyde (PFA) at room temperature for an hour, then immersed in 30% (w/v) sucrose until fully submerged before embedding and freezing in Optimal Cutting Temperature (OCT) compound. Using a cryostat at -20 °C, serial 12-μm sections were obtained from the embedded tissue. These sections were blocked with 5% normal donkey serum in PBST for 30 min and incubated with an anti-dystrophin antibody (1:500, ab15277, Abcam) diluted in blocking buffer overnight at 4 °C. Post-incubation, sections were treated with secondary goat anti-rabbit IgG Alexa Fluor 488 (1:400, A-11037, LifeTech) for an hour at room temperature, stained with 0.5 μg mL^{-1} Hoechst, and mounted. Photographs of the stained sections were taken under a Nikon Ti-S microscope, and Dystrophin-positive cells were quantified to assess restoration efficiency.

Histological Analysis

For histopathological examination, tibialis anterior muscle, liver, and stomach tissues were harvested from various groups of mice and fixed in 4% paraformaldehyde (PFA) for 48 hours. The

tissues were then subjected to sequential dehydration through ethanol gradients (70%, 95%, and 100%), cleared in xylene, and finally embedded in paraffin. Sections of 10-μm thickness were prepared and stained with Hematoxylin and Eosin (H&E) to assess tissue architecture and any pathological changes. The stained sections were scanned using a NanoZoomer Slice scanner, and the histological morphology was comparatively analyzed across the different mouse cohorts.

Grip Strength Test

The grip strength meter (Saiangsi) was used to measure the muscle strength of all four limbs in each group. Randomly picked mice from each group (wild-type C57BL/6, *mdx*, and *mdx* administered with NAEK or ChNAEK for 1, 2, and 4 weeks) were weighed and induced to grab the shelf connected to the meter by lifting them by the tail. The peak force in grams was recorded, with each mouse undergoing 4-6 trials and the average force noted. All measurements were performed blindly without knowledge of the group information.

Body Weight and Survival Rate

The body weight of each mouse was monitored daily throughout the experimental period. The survival rates of wild-type C57BL/6 mice orally administered with NAEK aqueous solution or ChNAEK formulation were observed, with untreated *mdx* mice serving as controls. This longitudinal tracking provided insights into the general health and potential systemic effects of the treatments.

Serum Biochemical Analysis

The serum biochemical components were analyzed by Soonbio technology. Blood samples were collected from the orbital vein of wild-type, *mdx*, NAEK-treated, and ChNAEK-treated *mdx* mice at 0, 1, 2, and 4 weeks. An automatic biochemical instrument (Beckman, AU480) measured the serum levels of creatine kinase, alanine aminotransferase, total cholesterol, total protein, albumin, urea, and creatinine. Three mice were included in each experimental cohort to ensure statistical robustness.

Supplementary figures

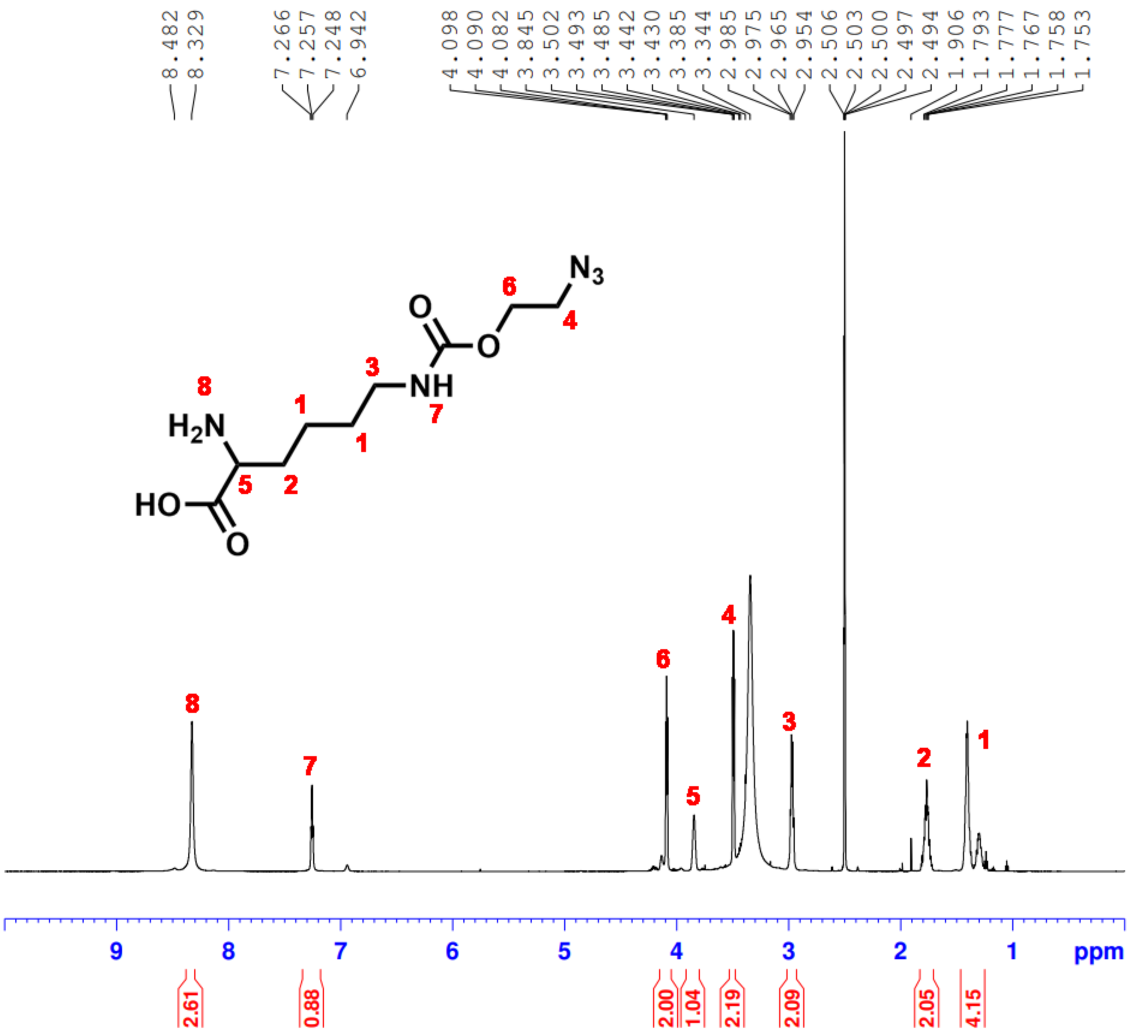

Figure S1. The ^{1}H NMR spectra of NAEK in DMSO-d6. ^{1}H NMR (600 MHz, DMSO-d6) δ 8.33 (s, 2H), 7.26 (t, J = 5.7 Hz, 1H), 4.16 – 4.04 (m, 2H), 3.85 (q, J = 6.4, 5.4 Hz, 1H), 3.52 – 3.47 (m, 2H), 2.97 (q, J = 6.4 Hz, 2H), 1.77 (tdd, J = 17.0, 12.6, 6.8 Hz, 2H), 1.44 – 1.26 (m, 4H).

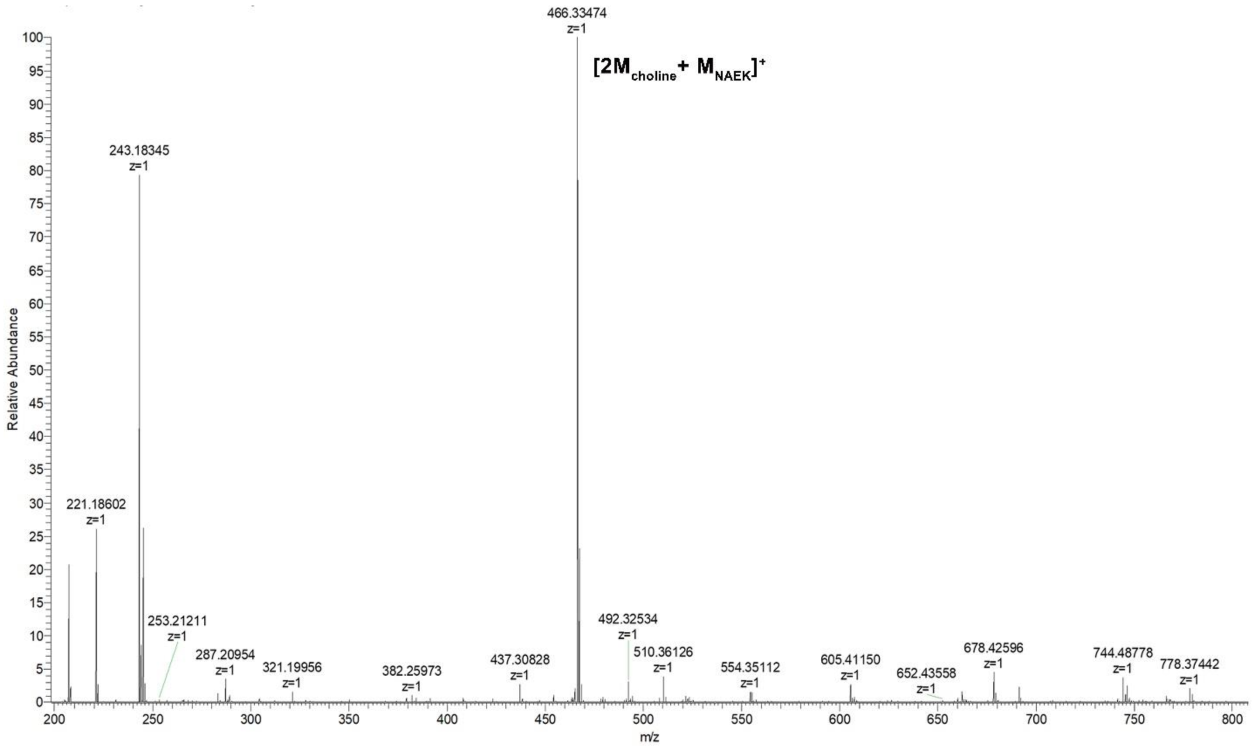

Figure S2 The Mass spectra of ChNAEK. The peak at 466 is due to the clusting of 2Choline 1NAEK. Clusters of the $[(AB)_nA]^+$ form consisting of cation A and anion B are reportedly one of the typical features of FAB-MS spectroscopy of ion beams at room temperature.

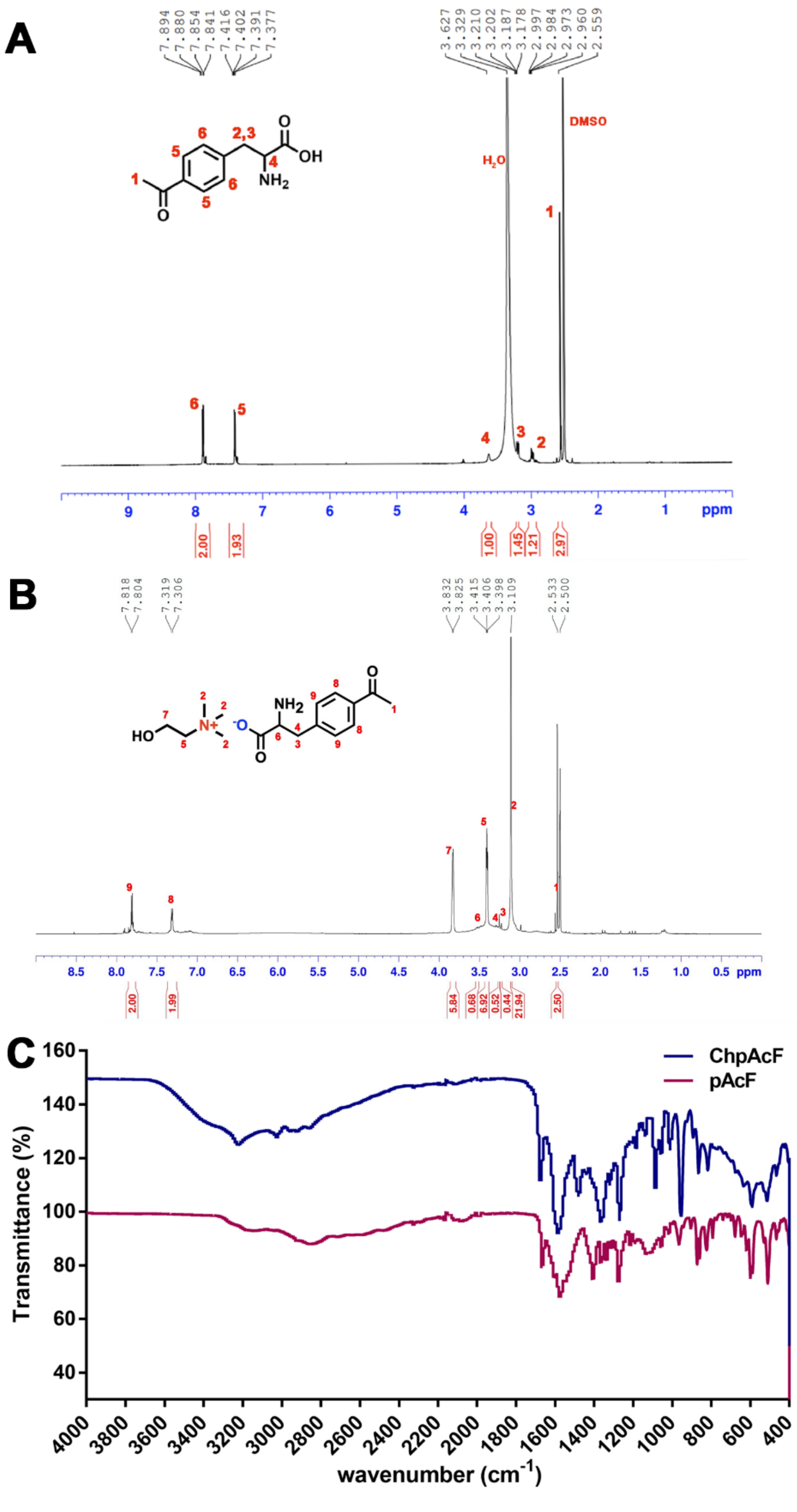

e Figure S3. Characterization of ChpAcF. (**A**) The ^{1}H NMR spectra of pAcF in DMSO-d6. pAcF: ^{1}H NMR (600 MHz, DMSO-d6) δ 7.91 – 7.83 (m, 2H), 7.43 – 7.35 (m, 2H), 3.63 (s, 1H), 3.19

(d, J = 9.1 Hz, 1H), 2.98 (dd, J = 14.2, 7.7 Hz, 1H), 2.55 (d, *J* = 6.5 Hz, 3H). (**B**) The ^{1}H NMR spectra of ChpAcF in DMSO-d6. ^{1}H NMR (600 MHz, DMSO-d6) δ 7.81 (dd, J = 8.1, 5.9 Hz, 2H), 7.31 (dd, J = 8.2, 2.9 Hz, 2H), 3.83 (td, J = 5.6, 2.7 Hz, 6H), 3.53 (s, 1H), 3.43 – 3.38 (m, 7H), 3.28 (s, 1H), 3.24 (d, J = 11.9 Hz, 1H), 2.57 – 2.51 (m, 3H). (**C**) The infrared spectrum of pAcF (Red) and ChpAcF (Blue). ChpAcF is a brownish yellow paste at room temperature or 37 °C, but it quickly transitioned into a liquid when the temperature rises to 50℃.

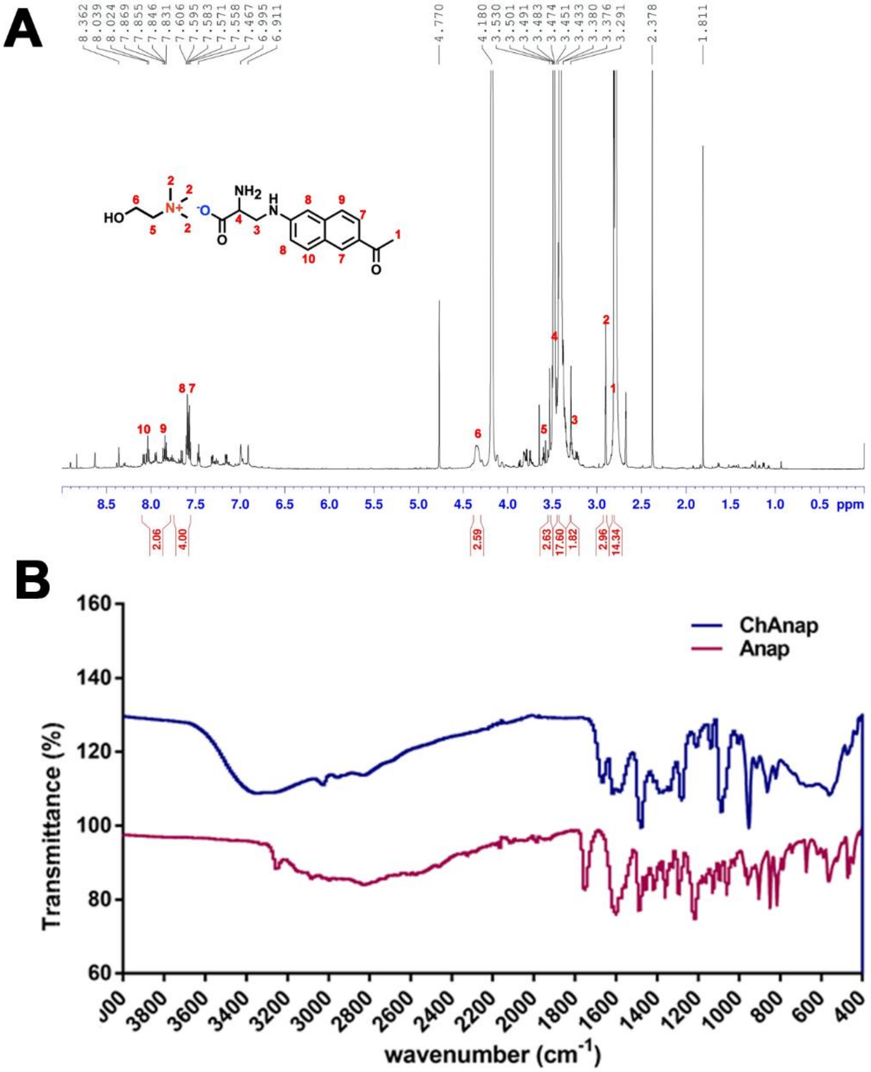

E

Figure S4. Characterization of ChAnap. (**A**) The ^{1}H NMR spectra of ChAnap in DMSO-d6. (**B**) The infrared spectrum of Anap (Red) and ChAnap (Blue). The resulting ChAnap is brown liquid at room temperature.

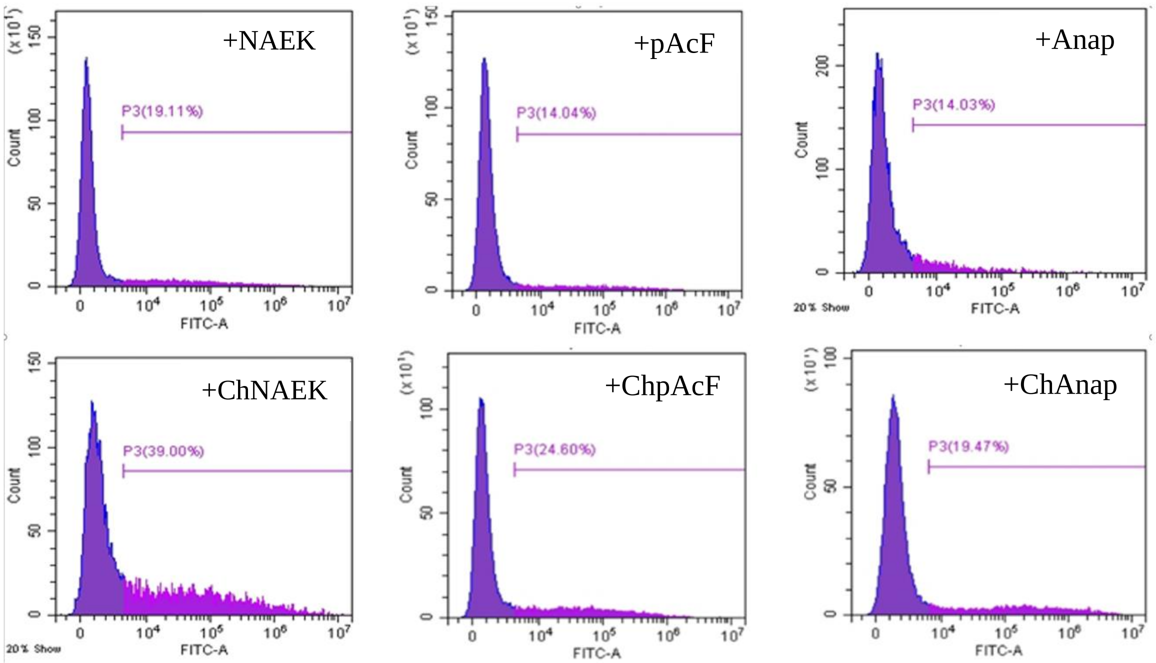

Figure S5. Effect of UAA-based ILs and UAAs on read-through rate of GFP^{39TAA} with the corresponding suppression system by fluorescence flow analysis (n=3)**.** The *y* axes represent the number of the cells, and the *x* axes shows the GFP fluorescence intensity (in arbitrary units). P3 represents the GFP restoration-positive cell percentage.

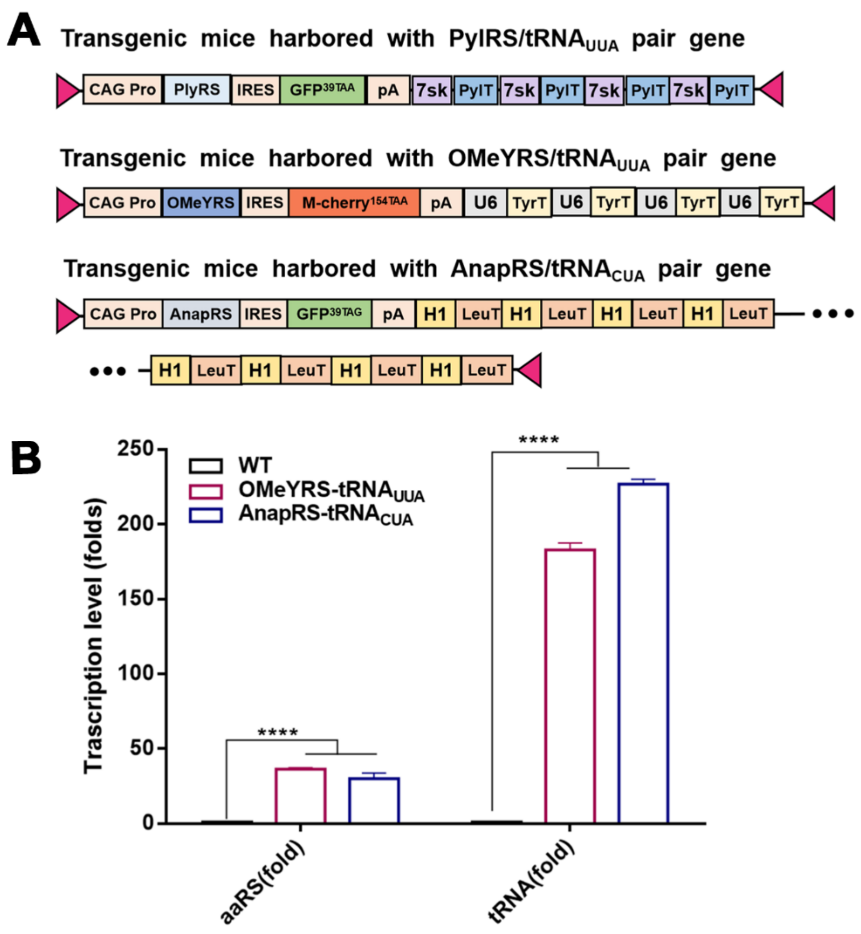

Figure S6. Construction and verification of the AnapRS/tRNA$_{CUA}$ transgenic mice. (**A**) The plasmids design of three orthogonal aaRS/tRNA pairs for the transgenic mice construction. (**B**)The transcription level of orthogonal aaRS and tRNA of the two transgenic mice compared with WT mice (*n*=3). The results were statistical analyzed using GraphPad Prism Software (Version 8.0, GraphPad Software, San Diego, CA) and presented as the mean ± S.D. **** $p < 0.0001$ one-way ANOVA followed with Tukey multiple comparisons tests.

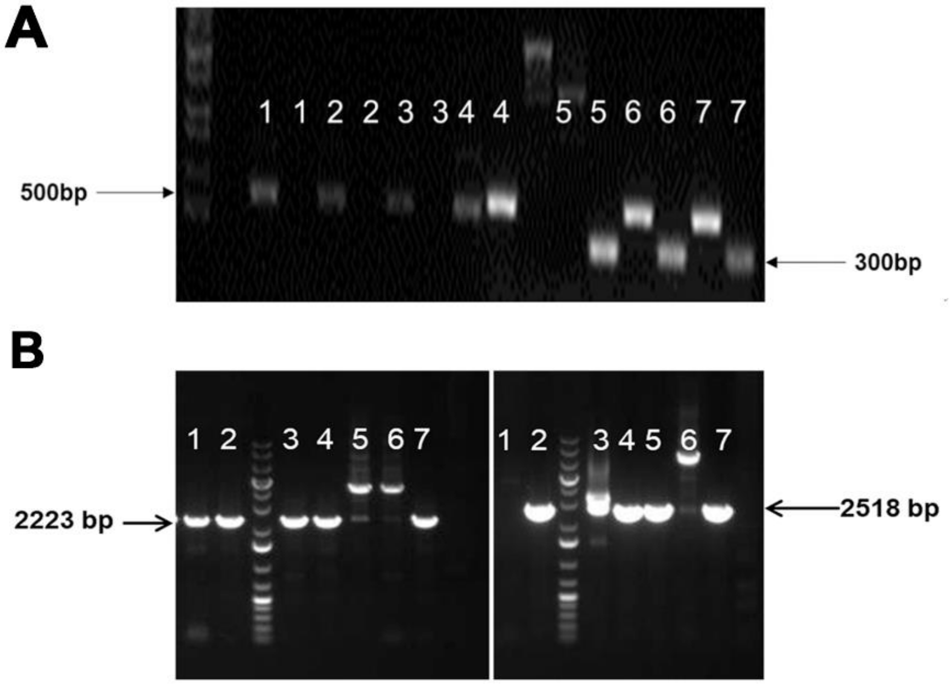

Figure S7. Verification of the AnapRS/tRNA$_{CUA}$ transgenic mice (**A**) **and OMeYRS/tRNA$_{UUA}$ transgenic mice** (**B**) **by PCR analysis of mouse tail DNA.** (**A**) Primer 1 targeting the sequence at 500 bp and Primer 2 targeting the sequence at 300 bp were used (*n*=3). The seven progenies were from the F1 generation. The results showed that Progeny 6 and 7 were positive. (**B**) Primer 1 targeting the sequence at 2223 bp and Primer 2 targeting the sequence at 2518 bp were used (*n*=3). The seven progenies were from the F1 generation. The results showed that Progeny 2, 4 and 7 were positive.

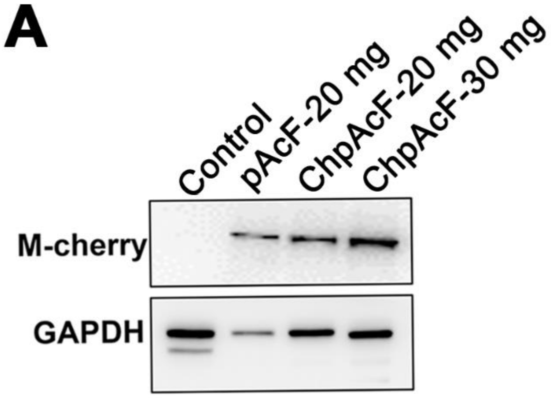

Figure S8. The western blot analysis of report protein in mice muscle to evaluate the restoration efficiency produced by the incorporation of UAAs. (**A**) The M-cherry^{154TAA} restoration of the transgenic mice harbored with OMeYRS-(U6-tRNA$_{UUA}$)$_4$-M-cherry^{154TAA} gene after intramuscular injection ChpAcF (the same molar dose of 20mg or 30 mg pAcF) and 20 mg pAcF aqueous suspention every day for 1 week. When the same dose of pAcF (20 mg) reached the local tissue, the mCherry expression of ChpAcF was significantly higher than that of the suspension group (*n*=2). (**B**) The GFP^{39TAG} restoration of the transgenic mice harbored with AnapRS-(H1-tRNA$_{CUA}$)$_8$-GFP^{39TAG} gene after intramuscular injection ChAnap (the same molar dose of 30 mg Anap) or 30 mg Anap aqueous suspention every day for 1 week (*n*=2). When the same dose of Anap (30 mg) reached the focal muscle tissue, the GFP expression of ChAnap was significantly higher than that of the suspension group. The bigger dose of UAA, the higher reporter protein restoration.

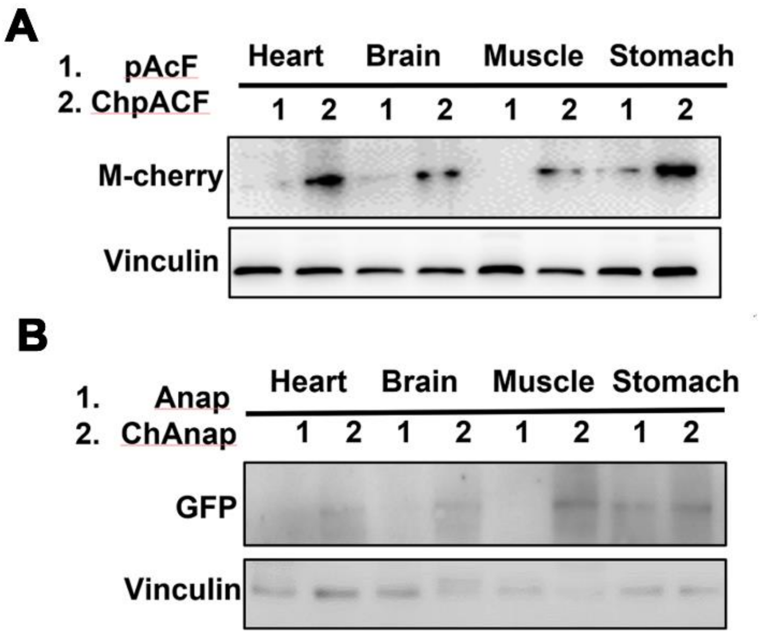

Figure S9. The western blot analysis of the report protein restoration in focal tissues of the transgenic mice after one-week oral treatment. (**A**) The M-cherry^{154TAA} restoration in heart, brain, muscle and stomach tissue of the transgenic mice treated with same molar dose of ChpAcF or pAcF aqueous suspension (30mg). (**B**) The GFP^{39TAG} restoration in heart, brain, muscle and stomach tissue of the transgenic mice treated with same molar dose of ChAnap or Anap aqueous suspension (20 mg). The results showed that the expression of M-cherry (pAcF groups) or GFP (Anap groups) in these focal tissues in UAA-based ILs groups were significantly higher than that of free UAA aqueous suspension groups, respectively, which were consistent with the trend of UAAs accumulation in tissues after oral administration.

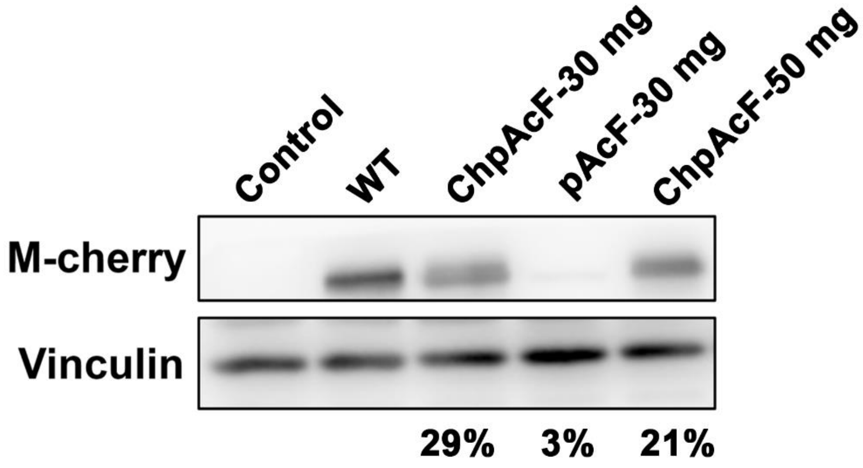

Figure S10. The efficiency of pAcF incorporation evaluated in muscle of the OMeYRS-tRNA-M-cherry^{154TAA} transgenic mice after oral administration by western blot analysis. ChpAcF (the same molar dose of 30 mg or 50 mg pAcF) or 30 mg pAcF aqueous suspension was given by gavage every day for 1 week (*n*=2). The integrated optical density (IOD) of GFP bands was normalized to that of the wild-type GFP after sample loading correction. Normalized IOD values are shown underneath the western blot. The results showed that 30mg was taken as the optimal oral dose of ChpAcF which resulted in almost the same M-cherry expression as 50 mg dose. When the same dose of pAcF (30 mg) was administered orally, the expression of M-cherry produced by ChpAcF (29%) was significantly higher than that of the suspension group (3%), which had almost no detectable protein expression.

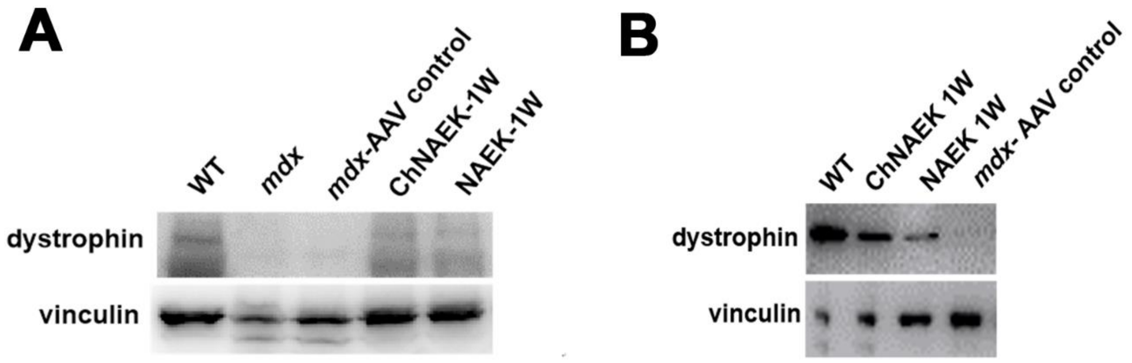

Figure S11. The western blot analysis of dystrophin restoration from the muscle of *mdx* mice after one-week injection treatment. (**A**) Same molar dose of ChNAEK or NAEK aqueous solution (20 mg) was injection intramuscularly every two days (*n*=2). (**B**) Same molar dose of ChNAEK or NAEK aqueous solution (30 mg) was injection intraperitoneally every two days (*n*=2).

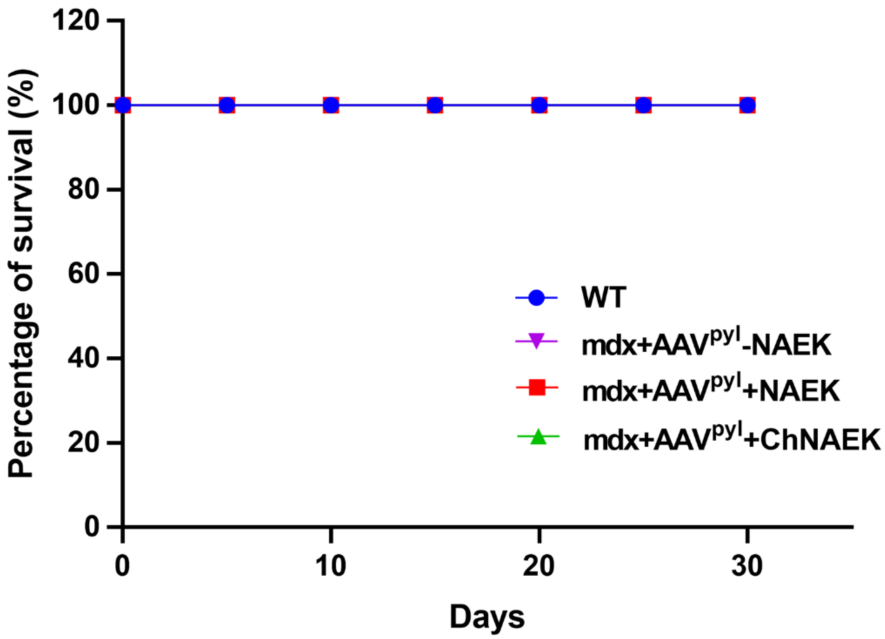

Figure S12. Survival rates of four different mice groups within 30 days (*n*=4)**.** The results were statistical analyzed using GraphPad Prism Software (Version 8.0, GraphPad Software, San Diego, CA) and presented as the mean ± S.D.

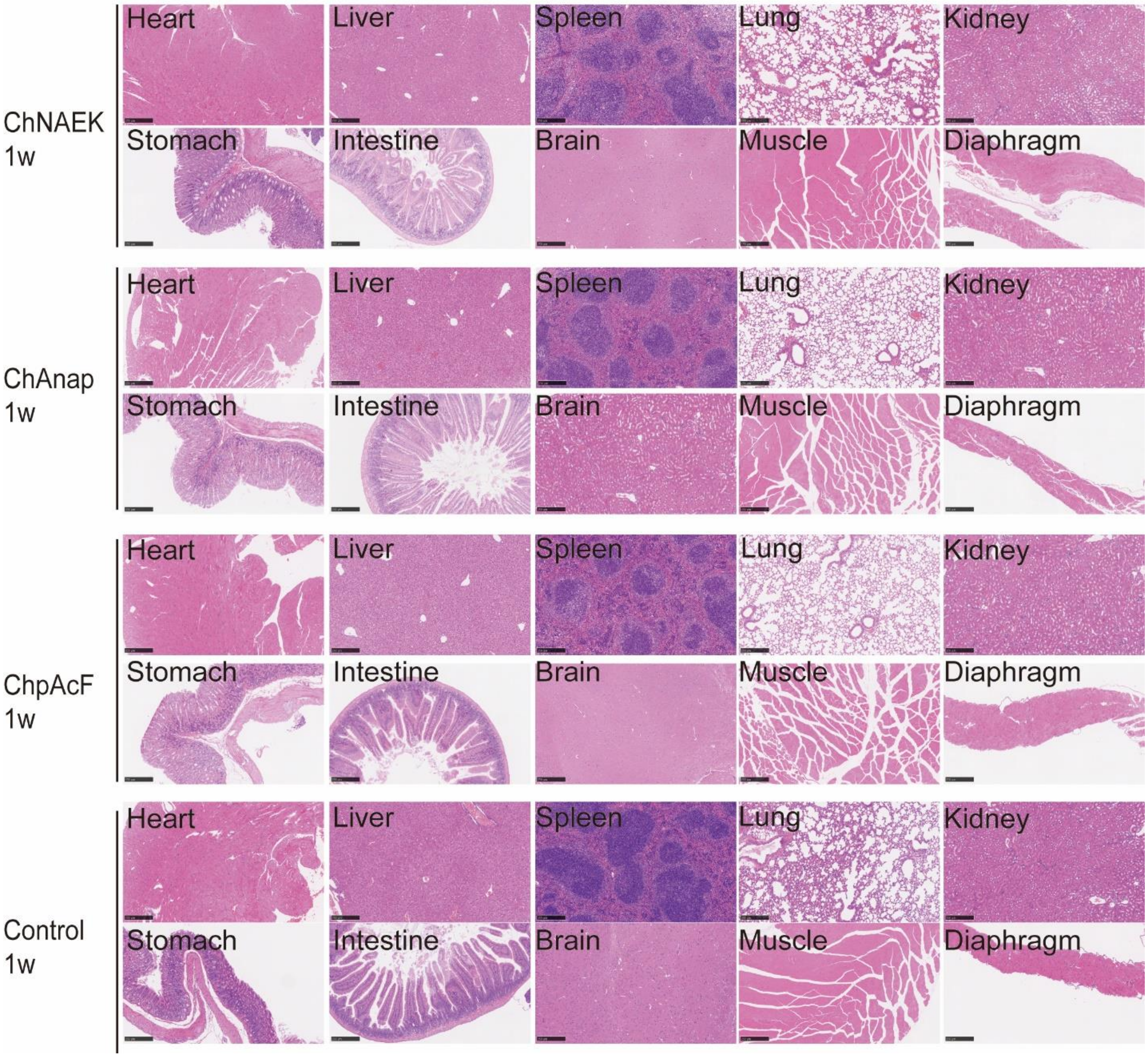

Figure S13 Different organ and tissue morphologies evaluated after 1 week of oral administration by H&E staining (*n*=3). Same molar dose of ChNAEK or NAEK aqueous solution (30 mg), ChpAcF or pAcF aqueous suspension (30 mg), ChAnap or Anap aqueous suspension (20 mg) was given by gavage every day for 1 week.

Supplementary tables

Table S1. Pharmacokinetic parameters of UAAs in mice serum after oral administration of UAAs or UAA-based ILs. *P < 0.05 vs UAA (mean±SD, *n* = 3)

Parameter/unit	NAEK	ChNAEK	pAcF	ChpAcF	Anap	ChAnap
AUC/μgL-1h-1	7.91±0.086	72.39±0.62	7.30±0.14	62.85±0.66	6.13±0.12	65.22±0.26
MRT/h	3.30±0.025	9.72±0.028	3.37±0.049	9.66±0.034	3.27±0.16	10.53±0.31
$T1_{/2}$/h	1.87±0.64	2.62±0.063	5.43±0.86	2.45±0.21	2.62±0.35	3.90±0.099
Tmax/h	2±0.00	8±0.00	2±0.00	9±0.00	2±0.00	8.33±0.58
C_{max}/μgL-1	1.89±0.040	7.07±0.053	1.63±0.081	6.40±0.12	1.59±0.055	5.88±0.046

Table S2. The primer sequences for qRT-PCR analysis.

PylRS-F	ATTGAAATGGCGTGTGGAGACC
PylRS-R	TGCCTTTGCAGAAACAGAATTTTCCA
$tRNA^{Pyl}$-F	CGGAAACCTGATCATGTAGATCG
$tRNA^{Pyl}$-R	ACCCCGGGAATCTAACCC
OMeYRS-F	GGAAGCGTTAGCAGAGCGAC
OMeYRS-R	GAACGGGGCAACCTGCTTAC
$tRNA^{Tyr}$-F	CTTGTGGAAAGGACGGTGGG
$tRNA^{Tyr}$-R	AATGGTGGGGGAAGGATTCG
AnapRS-F	AGACGAGAGCAAAGAGAAGTAT
AnapRS-R	GAGCTGGTTTTTCATATACGCGATG
$tRNA^{Leu}$-F	CCTATCAGTGATAGAGACACCG
$tRNA^{Leu}$-R	AAAATACCCGGAGCGGGACTT
GAPDH-F	CAATGTGTCCGTCGTGGATCT
GAPDH-R	GTCCTCAGTGTAGCCCAAGATG

Table S3. The primer sequences for the three kinds of transgenic mice tail DNA PCR analysis.

PylRS	For-1	CGTGTTCGTGCAAGTTGAGTCCATC
	Rev-1	TTTGATAAGGCTGCAGAAGGAGCGG
	For-2	CTACAGCTCCTGGGCAACGTG
	Rev-2	CAGCGTTTGCAGGTCTTCCTGTAT
OMeYRS	For-1	GGCGTTCAGGAAGATTATGGAGGGG
	Rev-1	AGTCCCTATTGGCGTTACTATGG
	For-2	GCTGGGGCTCAGATCTGTATAC
	Rev-2	TGGCTGCAGACTTAGCTTTCAGCTT
LeuRS	For-1	AGTCGCTCTGAGTTGTTATCAG
	Rev-1	TGAGCATGTCTTTAATCTACCTCGATG
	For-2	AGTCGCTCTGAGTTGTTATCAG
	Rev-2	AGTCCCTATTGGCGTTACTATGG

Other Sequences

PylRS

ATGGATAAAAAACCATTAGATGTTTTAATATCTGCGACCGGGCTCTGGATGTCCAGGACTGGCAC
GCTCCACAAAATCAAGCACCATGAGGTCTCAAGAAGTAAAATATACATTGAAATGGCGTGTGGA
GACCATCTTGTTGTGAATAATTCCAGGAGTTGTAGAACAGCCAGAGCATTCAGACATCATAAGTA
CAGAAAAACCTGCAAACGATGTAGGGTTTCGGACGAGGATATCAATAATTTTCTCACAAGATCA
ACCGAAAGCAAAAACAGTGTGAAAGTTAGGGTAGTTTCTGCTCCAAAGGTCAAAAAAGCTATGC
CGAAATCAGTTTCAAGGGCTCCGAAGCCTCTGGAAAATTCTGTTTCTGCAAAGGCATCAACGAAC
ACATCCAGATCTGTACCTTCGCCTGCAAAATCAACTCCAAATTCGTCTGTTCCCGCATCGGCTCCT
GCTCCTTCACTTACAAGAAGCCAGCTTGATAGGGTTGAGGCTCTCTTAAGTCCAGAGGATAAAAT
TTCTCTAAATATGGCAAAGCCTTTCAGGGAACTTGAGCCTGAACTTGTGACAAGAAGAAAAAAC
GATTTTCAGCGGCTCTATACCAATGATAGAGAAGACTACCTCGGTAAACTCGAACGTGATATTAC
GAAATTTTTCGTAGACCGGGGTTTTCTGGAGATAAAGTCTCCTATCCTTATTCCGGCGGAATACGT
GGAGAGAATGGGTATTAATAATGATACTGAACTTTCAAAACAGATCTTCCGGGTGGATAAAAAT
CTCTGCTTGAGGCCAATGCTTGCCCCGACTCTTTACAACTATCTGCGAAAACTCGATAGGATTTTA
CCAGGCCCAATAAAAATTTTCGAAGTCGGACCTTGTTACCGGAAAGAGTCTGACGGCAAAGAGC
ACCTGGAAGAATTTACTATGGTGAACTTCTGTCAGATGGGTTCGGGATGTACTCGGGAAAATCTT
GAAGCTCTCATCAAAGAGTTTCTGGACTATCTGGAAATCGACTTCGAAATCGTAGGAGATTCCTG
TATGGTCTTTGGGGATACTCTTGATATAATGCACGGGGACCTGGAGCTTTCTTCGGCAGTCGTCG
GGCCAGTTTCTCTTGATAGAGAATGGGGTATTGACAAACCATGGATAGGTGCAGGTTTTGGTCTT
GAACGCTTGCTCAAGGTTATGCACGGCTTTAAAAACATTAAGAGGGCATCAAGGTCCGAATCTTA
CTATAATGGGATTTCAACCAATCTGTAA

LeuRS

ATGGAAGAGCAATACCGCCCGGAAGAGATAGAATCCAAAGTACAGCTTCATTGGGATGAGAAGC
GCACATTTGAAGTAACCGAAGACGAGAGCAAAGAGAAGTATTACTGCTTTTCTGGCCCTCCCTAT
CCTTCTGGTCGACTACACATGGGCCACGTACGTAACTACACCATCGGTGACGTGATCGCCCGCTA
CCAGCGTATGCTGGGCAAAAACGTCCTGCAGCCGATCGGCTGGGACGCGTTTGGTCTGCCTGCGG
AAGGCGCGGCGGTGAAAAACAACACCGCTCCGGCACCGTGGACGTACGACAACATCGCGTATAT
GAAAAACCAGCTCAAAATGCTGGGCTTTGGTTATGACTGGAGCCGCGAGCTGGCAACCTGTACG
CCGGAATACTACCGTTGGGAACAGAAATTCTTCACCGAGCTGTATAAAAAAGGCCTGGTATATAA
GAAGACTTCTGCGGTCAACTGGTGTCCGAACGACCAGACCGTACTGGCGAACGAACAAGTTATC
GACGGCTGCTGCTGGCGCTGCGATACCAAAGTTGAACGTAAAGAGATCCCGCAGTGGTTTATCAA
AATCACTGCTTACGCTGACGAGCTGCTCAACGATCTGGATAAACTGGATCACTGGCCTGACACCG

TTAAAACCATGCAGCGTAACTGGATCGGTCGTTCCGAAGGCGTGGAGATCACCTTCAACGTTAAC
GACTATGACAACACGCTGACCGTTTACACTACCCGCCCGGACACCTTTATGGGTTGTACCTACCT
GGCGGTAGCTGCGGGTCATCCGCTGGCGCAGAAAGCGGCGGAAAATAATCCTGAACTGGCGGCC
TTTATTGACGAATGCCGTAACACCAAAGTTGCCGAAGCTGAAATGGCGACGATGGAGAAAAAG
GCGTCGATACTGGCTTTAAAGCGGTTCACCCATTAACGGGCGAAGAAATTCCCGTTTGGGCAGCA
AACTTCGTATTGATGGAGTACGGCACGGGCGCAGTTATGGCGGTACCGGGGCACGACCAGCGCG
ACTACGAGTTTGCCTCTAAATACGGCCTGAACATCAAACCGGTTATCCTGGCAGCTGACGGCTCT
GAGCCAGATCTTTCTCAGCAAGCCCTGACTGAAAAAGGCGTGCTGTTCAACTCTGGCGAGTTCAA
CGGTCTTGACCATGAAGCGGCCTTCAACGCCATCGCCGATAAACTGACTGCGATGGGCGTTGGCG
AGCGTAAAGTGAACTACCGCCTGCGCGACTGGGGTGTTTCCCGTCAGCGTTACTGGGGCGCGCCG
ATTCCGATGGTGACTCTAGAAGACGGTACCGTAATGCCGACCCCGGACGACCAGCTGCCGGTGAT
CCTGCCGGAGGATGTGGTAATGGACGGCATTACCAGCCCGATTAAAGCAGATCCGGAGTGGGCG
AAAACTACCGTTAACGGTATGCCAGCACTGCGTGAAACCGACACTTTCGACACCTTTATGGAGTC
CTCCTGGGTTCTTGCGCGCTACACTTGCCCGCAGTACAAAGAAGGTATGCTGGATTCCGAAGCGG
CTAACTACTGGCTGCCGGTGGATATCGCGATTGGTGGTATTGAACACGCCATTATGGAGAGTCTC
TACTGTCGCTTCTTCCACAAACTGATGCGTGATGCAGGCATGGTGAACTCTGACGAACCAGTTAA
ACAGTTGCTGTGTCAGGGTATGGTGCTGGCAGATGCCTTCTACTATGTTGGCGAAAACGGCGAAC
GTAACTGGGTTTCCCCGGTTGATGCTATCGTTGAACGTGACGAGAAAGGCCGTATCGTGAAAGCG
AAAGATGCGGCAGGCCATGAACTGGTTTATACCGGCATGAGCAAAATGTCCAAGTCGAAGAACA
ACGGTATCGACCCGCAGGTGATGGTTGAACGTTACGGCGCGGACACCGTTCGTCTGTTTATGATG
TTTGCTTCTCCGGCTGATATGACTCTCGAATGGCAGGAATCCGGTGTGGAAGGGGCTAACCGCTT
CCTGAAACGTGTCTGGAAACTGGTTTACGAGCACACAGCAAAAAGTGATGTTGCGGCACTGAAC
GTTGATGCGCTGACTGAAAATCAGAAAGCGCTGCGTCGCGATGTGCATAAAACGATCGCTAAAG
TGACCGATGATATCGGCCGTCGTCAGACCTTCAACACCGCAATTGCGGCGATTATGGAGCTGATG
AACAAACTGGCGAAAGCACCAACCGATGGCGAGCAGGATCGCGCTCTGATGCAGGAAGCACTGC
TGGCCGTTGTCCGTATGCTTAACCCGTTCACCCCGCACATCTGCTTCACGCTGTGGCAGGAACTGA
AAGGCGAAGGCGATATCGACAACGCGCCGTGGCCGGTTGCTGACGAAAAAGCGATGGTGGAAG
ACTCCACGCTGGTCGTGGTGCAGGTTAACGGTAAAGTCCGTGCCAAAATCACCGTTCCGGTGGAC
GCAACGGAAGAACAGGTTCGCGAACGTGCTGGCCAGGAACATCTGGTAGCAAAATATCTTGATG
GCGTTACTGTACGTAAAGTGATTTACGTACCAGGTAAACTCCTCAATCTGGTCGTTGGCGGGCCC
GTTTAA

OMeYRS

ATGGCAAGCAGTAACTTGATTAAACAATTGCAAGAGCGGGGGCTGGTAGCCCAGGTGACGGACG
AGGAAGCGTTAGCAGAGCGACTGGCGCAAGGCCCGATCGCACTCGTGTGTGGCTTCGATCCTACC

GCTGACAGCTTGCATTTGGGGCATCTTGTTCCATTGTTATGCCTGAAACGCTTCCAGCAGGCGGG
CCACAAGCCGGTTGCGCTGGTAGGCGGCGCGACGGGTCTGATTGGCGACCCGAGCTTCAAAGCT
GCCGAGCGTAAGCTGAACACCGAAGAAACTGTTCAGGAGTGGGTGGACAAAATCCGTAAGCAGG
TTGCCCCGTTCCTCGATTTCGACTGTGGAGAAAACTCTGCTATCGCGGCCAATAATTATGACTGGT
TCGGCAATATGAATGTGCTGACCTTCCTGCGCGATATTGGCAAACACTTCTCCGTTAACCAGATG
ATCAACAAAGAAGCGGTTAAGCAGCGTCTCAACCGTGAAGATCAGGGGATTTCGTTCACTGAGTT
TTCCTACAACCTGCTGCAGGGTTATAGTATGGCCTGTTTGAACAAACAGTACGGTGTGGTGCTGC
AAATTGGTGGTTCTGACCAGTGGGGTAACATCACTTCTGGTATCGACCTGACCCGTCGTCTGCAT
CAGAATCAGGTGTTTGGCCTGACCGTTCCGCTGATCACTAAAGCAGATGGCACCAAATTTGGTAA
AACTGAAGGCGGCGCAGTCTGGTTGGATCCGAAGAAAACCAGCCCGTACAAATTCTACCAGTTCT
GGATCAACACTGCGCGTGCCGACGTTTACCGCTTCCTGAAGTTCTTCACCTTTATGAGCATTGAAG
AGATCAACGCCCTGGAAGAAGAAGATAAAAACAGCGGTAAAGCACCGCGCGCCCAGTATGTACT
GGCGGAGCAGGTGACTCGTCTGGTTCACGGTGAAGAAGGTTTACAGGCGGCAAAACGTATTACC
GAATGCCTGTTCAGCGGTTCTTTGAGTGCGCTGAGTGAAGCGGACTTCGAACAGCTGGCGCAGGA
CGGCGTACCGATGGTTGAGATGGAAAAGGGCGCAGACCTGATGCAGGCACTGGTCGATTCTGAA
CTGCAACCTTCCCGTGGTCAGGCACGTAAAACTATCGCCTCCAATGCCATCACCATTAACGGTGA
AAAACAGTCCGATCCTGAATACTTCTTTAAAGAAGAAGATCGTCTGTTTGGTCGTTTTACCTTACT
GCGTCGCGGTAAAAAGAATTACTGTCTGATTTGCTGGAAAGGGCCCGTTTAA

tRNA1^{Pyl}

CACCGGAAACCTGATCATGTAGATCGAACGGACTTTAAATCCGTTCAGCCGGGTTAGATTCCCGG
GGTTTCCGTTTTTT

tRNA2^{Pyl}

CACCGGGGGGTGGATCGAATAGATCACACGGACTTTAAATCCGTGCAGGCGGGTGAAACTCCCG
CACCCCCCGTTTTTT

tRNA^{Leu}

GCCCGGATGGTGGAATCGGTAGACACAAGGGATTCTAAATCCCTCGGCGTTCGCGCTGTGCGGGT
TCAAGTCCCGCTCCGGGTA

tRNA1^{Tyr}

GGTGGGGTTCCCGAGCGGCCAAAGGGAGCAGACTTTAAATCTGCCGTCACAGACTTCGAAGGTT
CGAATCCTTCCCCCACCATTTTTT

tRNA2^{Tyr}

GGAGGGGTAGCGAAGTGGCTAAACGCGGCGGACTTTAAATCCGCTCCCTTTGGGTTCGGCGGTTC
GAATCCGTCCCCCTCCATTTTTT

CMV-promoter

GTGATGCGGTTTTGGCAGTACATCAATGGGCGTGGATAGCGGTTTGACTCACGGGGATTTCCAAG
TCTCCACCCCATTGACGTCAATGGGAGTTTGTTTTGGCACCAAAATCAACGGGACTTTCCAAAAT
GTCGTAACAACTCCGCCCCATTGACGCAAATGGGCGGTAGGCGTGTACGGTGGGAGGTCTATATA
AGCAGAGCT

7sk-promoter

CTGCAGTATTTAGCATGCCCCACCCATCTGCAAGGCATTCTGGATAGTGTCAAAACAGCCGGAAA
TCAAGTCCGTTTATCTCAAACTTTAGCATTTTGGGAATAAATGATATTTGCTATGCTGGTTAAATT
AGATTTTAGTTAAATTTCCTGCTGAAGCTCTAGTACGATAAGTAACTTGACCTAAGTGTAAAGTT
GAGATTTCCTTCAGGTTTATATAGCTTGTGCGCCGCCTGGGTACCTC

U6-promoter

GAGGGCCTATTTCCCATGATTCCTTCATATTTGCATATACGATACAAGGCTGTTAGAGAGATAATT
AGAATTAATTTGACTGTAAACACAAAGATATTAGTACAAAATACGTGACGTAGAAAGTAATAAT
TTCTTGGGTAGTTTGCAGTTTTAAAATTATGTTTTAAAATGGACTATCATATGCTTACCGTAACTT
GAAAGTATTTCGATTTCTTGGCTTTATATATCTTGTGGAAAGGAC

H1-promoter

TCGCTATGTGTTCTGGGAAATCACCATAAACGTGAAATCCCTATCAGTGATAGAGACTTATAAGT
TCCCTATCAGTGATAGAGA

Sequence of GFP

ATGAGTAAAGGAGAAGAACTTTTCACTGGAGTTGTCCCAATTCTTGTTGAATTAGATGGTGATGT
TAATGGGCACAAATTTTCTGTCAGTGGAGAGGGTGAAGGTGATGCAACATACGGAAAACTTACC
CTTAAATTTATTTGCACTACTGGAAAACTACCTGTTCCATGGCCAACACTTGTCACTACTTTCGGT
TATGGTGTTCAATGCTTTGCGAGATACCCAGATCATATGAAACAGCATGACTTTTTCAAGAGTGC
CATGCCTGAAGGTTATGTACAGGAAAGAACTATATTTTTCAAAGATGACGGGAACTACAAGACA
CGTGCTGAAGTCAAGTTTGAAGGTGATACCCTTGTTAATAGAATCGAGTTAAAAGGTATTGATTT

TAAAGAAGATGGAAACATTCTTGGACACAAATTGGAATACAACTATAACTCACACAATGTATAC
ATCATGGCAGACAAACAAAAGAATGGAATCAAAGTTAACTTCAAAATTAGACACAACATTGAAG
ATGGAAGCGTTCAACTAGCAGACCATTATCAACAAAATACTCCAATTGGCGATGGCCCTGTCCTT
TTACCAGACAACCATTACCTGTCCACACAATCTGCCCTTTCGAAAGATCCCAACGAAAAGAGAGA
CCACATGGTCCTTCTTGAGTTTGTAACAGCTGCTGGGATTACACATGGCATGGATGAACTATACA
AATAA

Sequence of M-cherry

ATGGTGAGCAAGGGCGAGGAGGACAACATGGCCATCATCAAGGAGTTCATGCGCTTCAAGGTGC
ACATGGAGGGCTCCGTGAACGGCCACGAGTTCGAGATCGAGGGCGAGGGCGAGGGCCGCCCCTA
CGAGGGCACCCAGACCGCCAAGCTGAAGGTGACCAAGGGCGGCCCCCTGCCCTTCGCCTGGGAC
ATCCTGTCCCCTCAGTTCATGTACGGCTCCAAGGCCTACGTGAAGCACCCCGCCGACATCCCCGA
CTACTTGAAGCTGTCCTTCCCCGAGGGCTTCAAGTGGGAGCGCGTGATGAACTTCGAGGACGGCG
GCGTGGTGACCGTGACCCAGGACTCCTCCCTGCAGGACGGCGAGTTCATCTACAAGGTGAAGCTG
CGCGGCACCAACTTCCCCTCCGACGGCCCCGTAATGCAGAAGAAGACCATGGGCTGGGAGGCCT
CCTCCGAGCGGATGTACCCCGAGGACGGCGCCCTGAAGGGCGAGATCAAGCAGAGGCTGAAGCT
GAAGGACGGCGGCCACTACGACGCCGAGGTCAAGACCACCTACAAGGCCAAGAAGCCCGTGCA
GCTGCCCGGCGCCTACAACGTCAACATCAAGCTGGACATCACCTCCCACAACGAGGACTACACC
ATCGTGGAACAGTACGAGCGCGCCGAGGGCCGCCACTCCACCGGCGGCATGGACGAGCTGTACA
AGTAA
